# Protein Kinase R Degradation Is Essential for Rift Valley Fever Virus Infection and Is Regulated by SKP1-CUL1-F-box (SCF)^FBXW11-NSs^ E3 Ligase

**DOI:** 10.1371/journal.ppat.1005437

**Published:** 2016-02-02

**Authors:** Rajini Mudhasani, Julie P. Tran, Cary Retterer, Krishna P. Kota, Chris A. Whitehouse, Sina Bavari

**Affiliations:** 1 Molecular and Translational Sciences Division, United States Army Medical Research Institute of Infectious Diseases, Frederick, Maryland, United States of America; 2 Perkin Elmer, Waltham, Massachusetts, United States of America; University of Texas Medical Branch at Galveston, UNITED STATES

## Abstract

Activated protein kinase R (PKR) plays a vital role in antiviral defense primarily by inhibiting protein synthesis and augmenting interferon responses. Many viral proteins have adopted unique strategies to counteract the deleterious effects of PKR. The NSs (Non-structural s) protein which is encoded by Rift Valley fever virus (RVFV) promotes early PKR proteasomal degradation through a previously undefined mechanism. In this study, we demonstrate that NSs carries out this activity by assembling the SCF (SKP1-CUL1-F-box)^FBXW11^ E3 ligase. NSs binds to the F-box protein, FBXW11, *via* the six amino acid sequence DDGFVE called the degron sequence and recruits PKR through an alternate binding site to the SCF^FBXW11^ E3 ligase. We further show that disrupting the assembly of the SCF^FBXW11-NSs^ E3 ligase with MLN4924 (a small molecule inhibitor of SCF E3 ligase activity) or NSs degron viral mutants or siRNA knockdown of FBXW11 can block PKR degradation. Surprisingly, under these conditions when PKR degradation was blocked, NSs was essential and sufficient to activate PKR causing potent inhibition of RVFV infection by suppressing viral protein synthesis. These antiviral effects were antagonized by the loss of PKR expression or with a NSs deleted mutant virus. Therefore, early PKR activation by disassembly of SCF^FBXW11-NSs^ E3 ligase is sufficient to inhibit RVFV infection. Furthermore, *FBXW11* and *BTRC* are the two homologues of the *βTrCP* (Beta-transducin repeat containing protein) gene that were previously described to be functionally redundant. However, in RVFV infection, among the two homologues of βTrCP, FBXW11 plays a dominant role in PKR degradation and is the limiting factor in the assembly of the SCF^FBXW11^ complex. Thus, FBXW11 serves as a master regulator of RVFV infection by promoting PKR degradation. Overall these findings provide new insights into NSs regulation of PKR activity and offer potential opportunities for therapeutic intervention of RVFV infection.

## Author Summary

Rift Valley fever (RVF) is a severe disease caused by infection with the Rift Valley fever virus (RVFV) that affects humans and livestock and occurs in large epidemics. Currently there are no FDA-approved drugs or vaccines to treat RVF. Many viruses have evolved unique strategies to overcome host immune responses in order to establish infection. One protein of RVFV called NSs is responsible for over-powering cellular antiviral defenses. NSs is known to degrade double-stranded (ds) RNA-dependent protein kinase (PKR), but neither the mechanism nor the functional significance of this activity has been fully understood. In this study we show that NSs promotes PKR degradation by recruiting PKR to the E3 ligase complex called SCF (SKP1-CUL1-F-box)^FBXW11^. A short stretch of six amino acids called the degron sequence in NSs regulates the NSs- FBXW11 interaction and is required for the assembly of the SCF^FBXW11^ complex. We further show that disruption of the SCF^FBXW11-NSs^ complex, with either a small molecule or with NSs degron viral mutants, can block PKR degradation. Surprisingly, when NSs mediated PKR degradation was blocked, NSs was essential and sufficient to activate PKR, causing potent inhibition of RVFV infection by suppressing viral protein synthesis. Therefore early PKR activation induced by inactivation of the SCF^FBXW11^ is sufficient to induce potent inhibition of RVFV infection. These findings may provide new molecular targets for therapeutic intervention of this important disease.

## Introduction

Activated double-stranded (ds) RNA-dependent protein kinase (PKR or EIF2AK2) plays a vital role in antiviral defense primarily by inhibiting protein synthesis and augmenting interferon responses [1]. PKR is a serine/threonine kinase that is maintained as an inactive monomer and undergoes activation in response to dsRNA and/or cellular stress signals, primarily resulting from viral infection. Activated PKR undergoes auto-phosphorylation and inhibits protein synthesis by phosphorylation of the eIF2-α (eukaryotic translation initiation factor 2 subunit alpha or EIF2A). The importance of PKR in innate antiviral responses is suggested by the existence of a multitude of viral regulators of PKR action. Proteasomal degradation of PKR is one of the numerous strategies used by viruses to impair PKR function.

Most of the targeted protein ubiquitination and subsequent proteasomal degradation are achieved by Cullin-RING E3 ligases (CRL [2,3]). The CRLs are modular assemblies centered on one of the several cullin scaffolds CUL1, CUL2, CUL3, CUL4A, CUL4B, CUL5, CUL7 and CUL9 forming the corresponding CRL1, CRL2, CRL3, CRL4A, CRL4B, CRL5, CRL7 and CRL9 ligases. The C terminal domain (CTD) of the cullin module contains an embedded RING finger protein (RBX1 or RBX2) that serves as the site for E2 binding and ubiquitin transfer activity. The amino terminus has an adaptor protein that binds to substrate receptors to recruit specific target proteins destined for ubiquitination. The SCF E3 ligase or CRL1, consisting of SKP1, CUL1, and an F-box protein (SCF), is the founding member of the CRLs. The substrate specificity is determined by the adaptor protein SKP1 and any of the 72 F-box substrate receptors bound to the N-terminus of CUL1 module.

The enzymatic activity of CRLs is dependent on cullin modification by the covalent attachment of NEDD8, a 9-kDa ubiquitin-like molecule, which requires the activity of the NEDD8 activating enzyme, NAE1[3]. Recently, a specific NAE1 small molecule inhibitor, MLN4924, has been developed and is currently in clinical trials for cancer therapy [4]. MLN4924 inhibition of cullin NEDDylation blocks CRL activity resulting in the accumulation of CRL targets.

Rift Valley fever virus (RVFV) is a member of the genus *Phlebovirus* within the family *Bunyaviridae*. This virus causes a severe disease, Rift Valley fever (RVF), that affects humans and livestock throughout Africa and the Arabian Peninsula. RVF occurs in large epidemics affecting public health and agriculture resulting in significant economic losses. Currently, there are no FDA approved drugs or vaccines to treat RVF. Understanding the molecular mechanisms of RVFV infection may lead to the development of new therapeutics. The RVFV genome is composed of three RNA segments, the S-, M- and L-segments, which code for the viral structural proteins, nucleocapsid (N) protein that encapsidates the viral RNA to form ribonuceloprotein (RNPs) complexes, the envelope glycoproteins (Gn and Gc), and the RNA-dependent RNA polymerase (RdRp), respectively. Virus entry into cells is mediated by the binding of the envelope glycoproteins (Gn/Gc) to an unknown cell surface receptor which mediates virus endocytosis. Acidification of the virus-containing endocytotic vesicle promotes virus-host membrane fusion and results in the release of the RNPs and RdRp into the cytoplasm, where transcription and replication of the viral genome occurs [5]. The glycoproteins Gn and Gc are produced by a glycoprotein precursor (GPC) that is co-translationally cleaved by an unknown protease. Gn and Gc form heteromeric complex which localize in steady-state at the Golgi apparatus where multiple interactions of the glycoproteins with RNPs and RdRp are believed to cause a change in membrane curvature leading to virus budding into the Golgi lumen [6–8]. Release of the virus-filled vesicles from the Golgi and their subsequent fusion with cell plasma membrane results in the release of mature virions into the extracellular medium. Thus, glycoprotein (Gn/Gc) expression on the cell surface reflects successful completion of the virus life cycle that spans approximately 10–12 h [9].

The viral genome also encodes three nonstructural proteins, NSs, NSm (NSm2) and the less characterized 78-kD NSm1. NSs is considered the major virulence factor of RVFV [10]. For instance, the C13 strain of RVFV, which has an in-frame deletion of the majority of the NSs gene, is avirulent in mice and sheep [11,12]. NSs effectively blocks antiviral responses by inhibiting both interferon synthesis and further downstream transcription of interferon-induced genes. NSs also promotes PKR proteasomal degradation during early stages of virus infection [13,14]. Recent evidence suggests that PKR degradation is required for efficient viral replication [15]; however, the molecular mechanisms regulating the NSs-mediated PKR proteasomal degradation have not been well-characterized.

We sought to investigate the molecular mechanism of PKR degradation and found that early PKR degradation is essential for RVFV infection. NSs targets PKR degradation by assembling (SKP1-CUL1-F-box) SCF^FBXW11^ E3 ligase *via* its binding to F-box protein FBXW11. A degron sequence, DDGFVE_263_, in NSs protein regulates the NSs-FBXW11 interaction and the SCF E3 ligase assembly. Disrupting the assembly of the SCF^FBXW11-NSs^ with the small molecule MLN4924 or RVFV encoding NSs degron mutants or siRNA knockdown of FBXW11 successfully blocks PKR degradation. Under these disruptive conditions, NSs expression was sufficient for early PKR activation, causing potent inhibition of RVFV infection by suppressing protein synthesis. Furthermore, FBXW11 is the limiting factor in SCF^FBXW11-NSs^ assembly and thereby serves as a master regulator of RVFV infection. Overall, these findings illustrate that inactivation of SCF^FBXW11-NSs^ is sufficient to induce early PKR activation and inhibition of RVFV infection.

## Results

### MLN4924 inhibits RVFV infection

**Fig 1:**
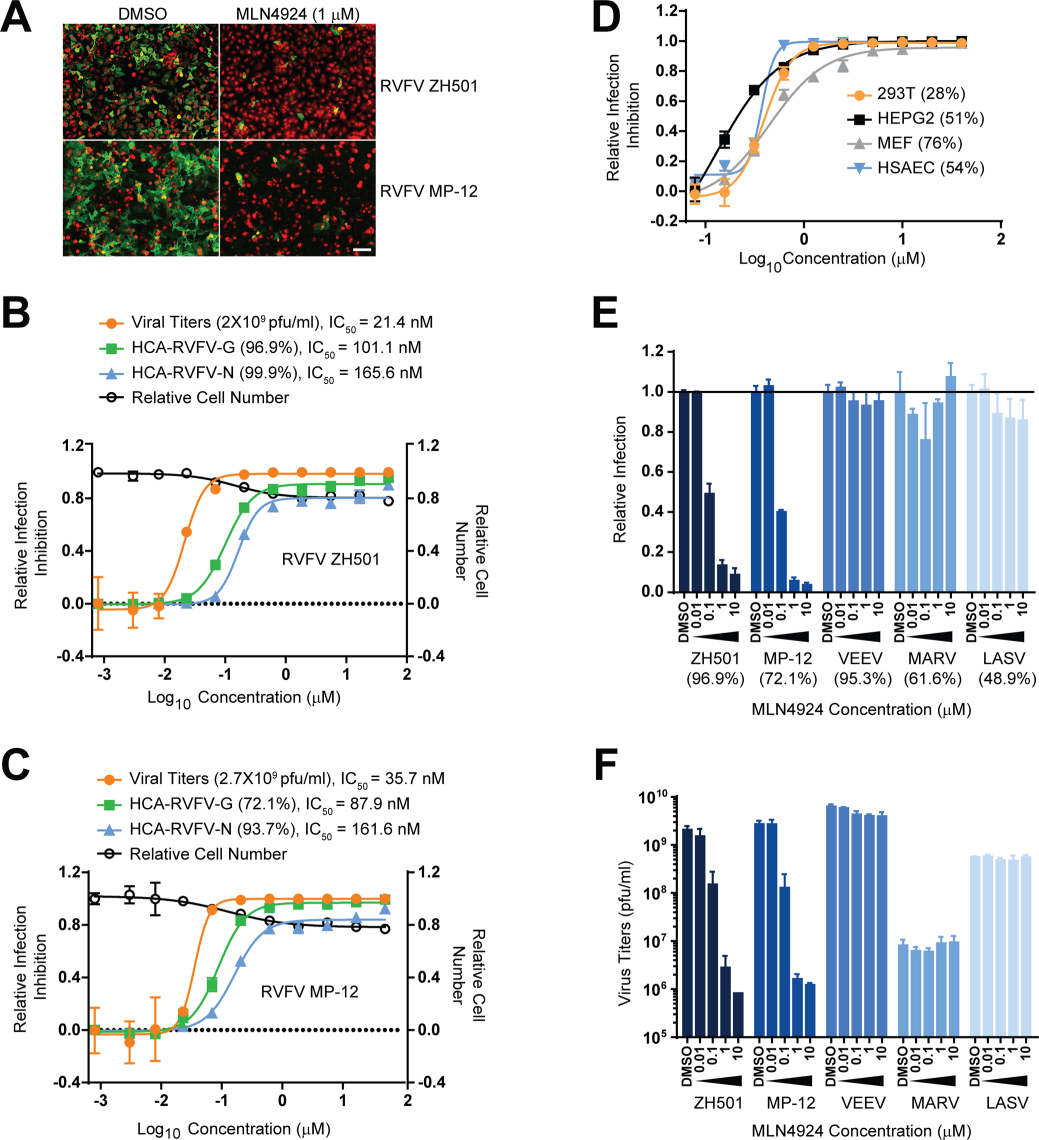
MLN4924 inhibits RVFV infection. **(A)** IFA of RVFV N or G expression (green) in HeLa cells (cytoplasm in red) that were infected with ZH501 (MOI = 1, 24h) or MP-12 (MOI = 1, 24h) and were either treated with control DMSO (0.5%) or MLN4924 (1 μM) from 1h prior to start of infection to the end of the infection. **(B-C)** Dose response curve analyses of MLN4924’s antiviral activity against ZH501 (B) and MP-12 (C) virus infection in HeLa cells. Virus infections were measured by HCA of G or N expressing cells or by plaque assay. The values in the brackets next to the name of the dose response curve indicates the average infection in mock (DMSO) treated cells. The infection rates of MLN4924 treated cells were normalized with the corresponding values derived from DMSO treated controls, which were considered as 100% and expressed as the mean ± SD. The final values were expressed as the mean ± SD. **(D)** Dose response curve analyses of the MLN4924’s antiviral activity against RVFV ZH501 in different cell lines. Infections were measured by HCA of N expressing cells. The values in the brackets next to the names of each cell line, indicate the average infection rate of virus infected cells that were mock (DMSO) treated. Data were normalized as in B-C **(E-F)** MLN4924 activity against different viruses: VEEV (MOI = 0.1, 24h), MARV (MOI = 3, 48h) and LASV (MOI = 1, 48h) when compared to RVFV ZH501 and RVFV MP-12 infections in HeLa cells. The virus infections were measured by HCA of viral antigen expressing cells (E) or by plaque assay (F): the relative infection in E was calculated by normalizing infection rates of compound treated cells with mock treated cells. The values in the brackets below the virus names indicate the average infection rate in mock (DMSO) treated cells.

To explore the mechanism of NSs mediated PKR proteasomal degradation, we first examined how small molecule inhibitors targeting different stages of the ubiquitin (UB)-proteasome pathway (UPP), including MG132 (proteasome inhibitor) and MLN4924 (CRL E3 ligase inhibitor), regulated RVFV infection. Both MG132 and MLN4924 inhibited RVFV infection, but to gain mechanistic insights into this inhibition, we focused on MLN4924 since it targets a specific class of E3 ligases unlike MG132, which globally inhibits the UPP. As shown in Fig 1A, MLN4924 at 1 μM inhibits the viral antigen expression of both the virulent strain ZH501 and the vaccine strain MP-12 based on immunofluorescence analysis (IFA) of HeLa cells. The latter were infected with the corresponding viruses for 24h at an MOI (multiplicity of infection) = 1. The potency of MLN4924 was determined by a 10-point concentration response curve in RVFV-infected HeLa cells (Fig 1B and 1C). Viral infections were measured by high-content image based analysis (HCA) using previously described methods [16,17]. Briefly, images of immunofluorescently labeled viral antigen-expressing cells were acquired by automated high-speed microscopy and subjected to image analysis software to enumerate the viral antigen expressing cells. The assay conditions were optimized such that greater than 50% of cells expressed viral antigen after multiple rounds of infection at a minimal MOI (further details in Methods section). HeLa cells treated with increasing concentrations of MLN4924 were infected with either ZH501 (Fig 1B) or MP-12 (Fig 1C) in a 96-well plate at an MOI = 1. After 24h, cells were subjected to IFA of glycoprotein (G) or nucleocapsid (N) protein expression. Viral infection was measured by HCA of N or G expression and was also compared to viral titers determined by plaque assay of the cell supernatants (Fig 1B and 1C). As shown in Fig 1B and 1C, irrespective of the method used to measure viral infection, MLN4924 showed a dose dependent inhibition of RVFV infection. Furthermore IC_50_ values (of 10 nM—150 nM) fall within the range of values expected for MLN4924’s activity on CRL in cell culture assays [4] and there was no significant decrease in cell number when compared to mock-treated cells for the duration of the infection (24h). The IC_50_ values by plaque assay were lower than the IC_50_ values by HCA due to the differences in the parameters measured, but nevertheless followed the same trend of inhibition. For example when the percentage of G expressing cells decreased by two fold, the corresponding virus titers dropped by a log value (Fig 1B and 1C). For rest of the study, HCA was used for measuring RVFV infection. Under the conditions of infection described for HCA, RVFV infection showed a linear increase in percentage of antigen expressing cells with increasing MOI (S1A and S1B Fig). A similar trend in the increase of viral titers was observed when viral infection was measured by plaque assay; however, HCA is relatively more sensitive to small changes in MOI and is a more quantitative and robust assay compared to the plaque assay (S1A and S1B Fig).

The MLN4924 mediated RVFV infection inhibition was also observed in multiple primary and cancer cell lines of human or mouse origin (Fig 1D). MLN4924 antiviral activity was specific to RVFV and was not observed with other RNA viruses including Venezuelan equine encephalomyelitis virus (VEEV), Lake Victoria Marburg virus (MARV), or Lassa virus (LASV) within the families *Togaviridae*, *Filoviridae*, and *Arenaviridae*, respectively, when viral infections were measured either by HCA (Fig 1E) or plaque assay (Fig 1F).

MLN4924 inhibits CRL activity by blocking conjugation of NEDD8 to the Cullin subunit. Further genetic evidence of NEDDylation’s role in RVFV infection was determined by overexpression of a point mutant, UBC12 (C111S), in the E2 NEDD8-conjugating enzyme, which has a dominant-negative effect on NEDD8 conjugation [18]. UBC12 (C111S) expression, and not the empty vector, inhibited RVFV infection specifically thus providing genetic proof for the role of NEDDylation (S1C Fig).

We next examined the stage of the viral life cycle that is critically dependent on CRLs by time of compound addition (ToA) studies (S1D Fig) and by viral RNA and gene expression kinetics during one viral replication cycle, which is ~12h (S1E–S1G Fig). HeLa cells were infected with a high MOI (MOI = 10) to achieve an infection rate >80% during one cycle of viral replication. Infection rates in ToA were evaluated by HCA of G expression in non-permeabilized cells in order to detect virus egress. ToA studies showed that MLN4924 treatment could be postponed until 5h post infection (PI [S1D Fig]). The kinetics of early N and late G gene expression in permeabilized cells showed that late gene expression was severely inhibited, while early gene expression was normal until 6h PI (S1E Fig). The inhibition in viral RNA expression levels also began to appear from 5-7h PI (S1F Fig) and compound addition could be postponed until 5h PI (S1G Fig). Collectively, these data suggest that MLN4924 does not target viral entry, rather it inhibits viral RNA and gene expression beginning at 5-6h PI as late gene expression was more severely inhibited than the early gene.

### MLN4924 causes NSs-dependent early PKR activation in RVFV-infected cells

**Fig 2:**
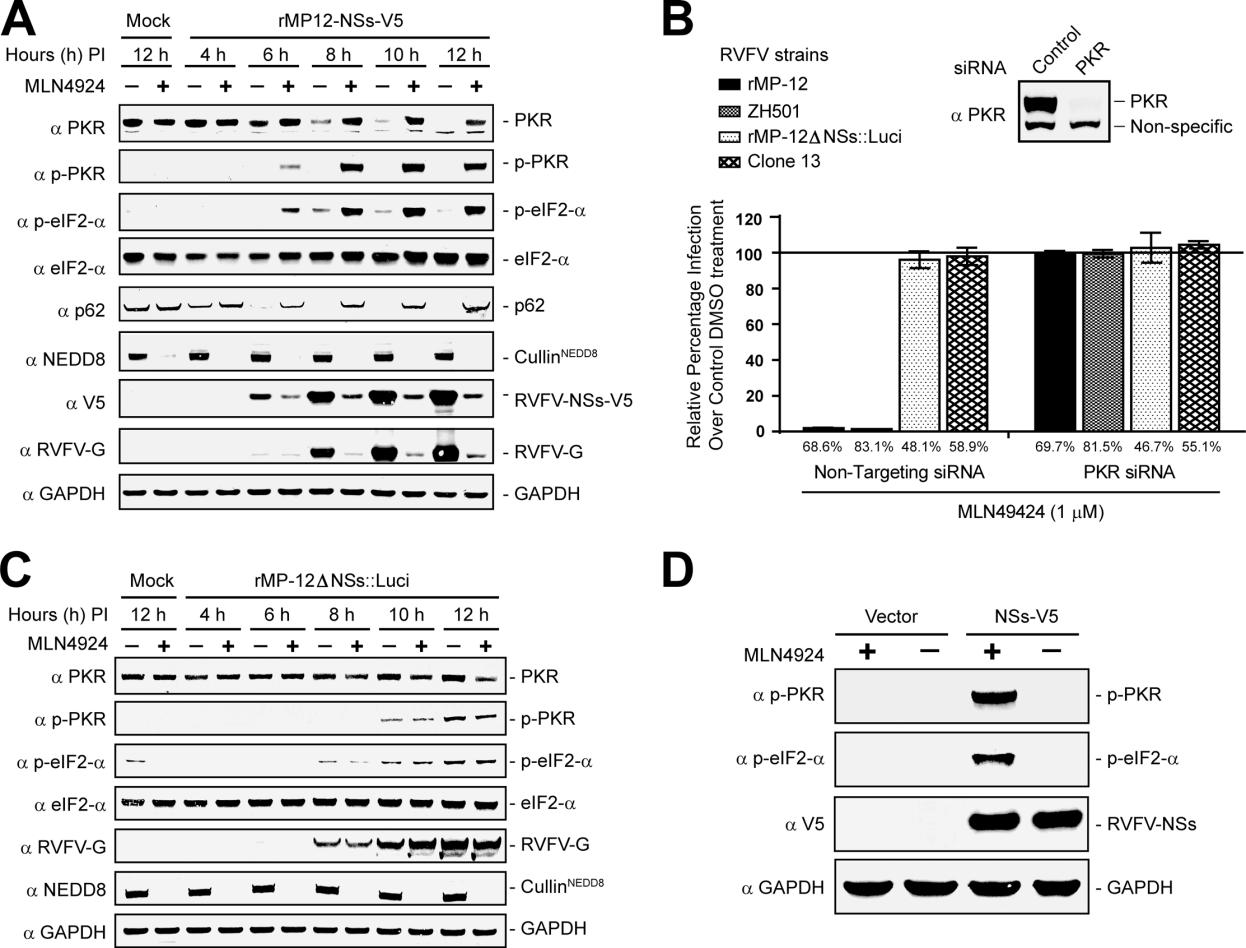
MLN4924 regulates NSs-dependent early PKR activation in RVFV-infected cells. **(A)** Western blot analysis demonstrating MLN4924 regulation of PKR activation and inhibition of viral gene expression during the course of a single cycle of viral replication (~ 12h). HeLa cells treated with control (DMSO) or MLN4924 (1 μM) were either mock infected or infected with rMP12-NSs-V5. At the indicated time points, cell lysates were harvested and analyzed for gene expression by Western blot analysis. **(B)** NSs regulates MLN4924 mediated PKR activation during RVFV infection. HeLa cells expressing control non-targeting siRNA or PKR siRNA were treated with DMSO (vehicle control) or MLN4924 (1 μM) and infected with either wildtype viruses including RVFV ZH501 or rMP-12 or NSs deficient viruses including clone 13 or rMP-12ΔNSs::Luci at an MOI = 10 for 12h. The infection rates were determined by enumerating G expressing cells (rMP-12 and rMP-12ΔNSs::Luci) or N expressing cells (RVFV ZH501 and clone13) by HCA. The data derived from MLN4924 treated cells was normalized with the corresponding DMSO treated controls (average infection rates are indicated under the bar graph), which were considered as 100% and expressed as the mean ± SD. The inset shows a decrease in PKR expression levels in PKR siRNA transfected cells by Western blot analysis. **(C)** Western blot analysis demonstrating the regulation of the PKR activation pathway and viral gene expression kinetics by NSs deficient virus (rMP-12ΔNSs::Luci virus) during single cycle of virus infection. (**D**) Western blot analysis which shows that MLN4924 treatment of HeLa cells that were transiently transfected with plasmid DNA expressing the NSs gene was sufficient to activate PKR.

Since MNL4924 regulated CRL E3 ligase activity, we hypothesized that MLN4924 treatment of RVFV infected cells might block NSs-mediated PKR proteasomal degradation resulting in accumulation of activated and phosphorylated PKR (p-PKR), which then might phosphorylate eIF2-α (p-eIF2-α) to suppress further viral protein synthesis. Thus, we examined the kinetics of PKR, p-PKR and p-eIF2-α expression during the course of a single cycle of infection (i.e., 12 h) in DMSO (vehicle control) or MLN4924 treated HeLa cells (Fig 2A). Due to a lack of suitable antibodies to the NSs protein, recombinant MP-12 (rMP-12) virus containing the NSs gene engineered with a V5 tag at the C terminus *i*.*e*., rMP-12-NSs-V5, was used in place of wildtype MP-12. As shown in Fig 2A, HeLa cells infected with rMP-12-NSs-V5 and treated with MLN4924 failed to clear PKR and led to a robust and simultaneous induction of p-PKR and p-eIF2-α beginning at 6h PI. Induction of p-PKR coincided with NSs expression. As expected, phosphorylation of eIF2-α suppressed further increases in NSs levels or synthesis of the late viral G protein, whose expression normally follows NSs. MLN4924 treatment also restored p62 expression levels, which under normal infection conditions would be targeted by NSs for proteasomal degradation [19]. As a control for MLN4924 activity, the lysates were also probed with anti-NEDD8 antibodies to determine the status of cullin NEDDylation. As expected, control treated cells showed a strong signal at a molecular weight that corresponded to that of cullin proteins, while no bands were detected with the MLN4924 treated lysates (Fig 2A). Collectively, these data show that MLN4924 prevented PKR degradation and instead activated PKR.

The above data suggested that NSs probably blocked PKR activation by depleting PKR from cells. If that was the case, NSs deficient virus might induce PKR activation. Furthermore, if MLN4924’s antiviral activity was primarily due to PKR activation, loss of PKR expression should restore RVFV infection. Both these hypotheses were tested by measuring infection rates of wildtype or NSs-deficient RVFV mutants treated with DMSO (vehicle control) or MLN4924 under normal or low PKR siRNA knockdown conditions during one cycle of virus replication (Fig 2B). Two different NSs deficient mutants were used: Clone-13, which is a naturally attenuated isolate of RVFV encoding a non-functional NSs gene due to a large in-frame deletion, and rMP-12ΔNSs::Luci virus, in which the NSs gene of MP-12 is replaced with a luciferase (Luci) gene by reverse genetics [20]. We confirmed that PKR expression levels were indeed reduced in HeLa cells transfected with siRNA targeting PKR (inset in Fig 2B). MLN4924 treatment inhibited MP-12 and ZH501 infection and as predicted this inhibition was completely rescued in cells with low levels of PKR expression (Fig 2B). Therefore PKR activation was responsible for MLN4924 antiviral activity. But surprisingly, NSs deficient viruses (clone 13 and rMP-12ΔNSs::Luci) were insensitive to MLN4924 treatment. In addition, replication of these viruses did not increase under low PKR expression levels. One explanation was that NSs was essential for early PKR activation; alternatively, it was also possible that the NSs deficient viruses had delayed replication kinetics that may have further delayed PKR activation.

To address these questions, we examined the kinetics of viral gene expression and the PKR activation pathway by Western blot analysis during one cycle of virus replication with the NSs deficient virus (rMP-12ΔNSs::Luci) under mock or MLN4924 treatment conditions (Fig 2C). This virus showed the same kinetics of G expression as wildtype rMP-12, with an increase in G expression becoming apparent by 8 h PI in both viruses (compare G expression kinetics in Fig 2A and 2C). These data suggested that at least during the early stages of the first cycle of viral replication there is no significant difference in the replication kinetics between NSs deficient virus when compared to rMP-12 virus. In agreement with previous observations (Fig 2B), the NSs deficient virus was insensitive to MLN4924 treatment as no decrease in G expression levels during MLN4924 treatment was observed. Moreover, MLN4924 did not induce early PKR activation. However, the NSs deficient virus did induce late PKR activation at 10–12 h PI, by which time most of the viral proteins were expressed and the virus egress had already been initiated. Thus, NSs is essential for early PKR activation upon MLN4924 treatment in RVFV infected cells.

We next examined if NSs is sufficient for PKR activation by transient transfection of NSs into HeLa cells that were treated with DMSO (vehicle control) or MLN4924 (Fig 2D). Western blot analysis showed that NSs expression alone was sufficient to induce PKR activation (based on increase in p-PKR and p-eIF2-α expression levels) by MLN4924 treatment. Collectively, these data suggest that NSs has dual antagonistic functions, promoting PKR degradation, but also activating PKR under conditions when NSs-mediated PKR degradation is blocked. We next sought out to identify the specific CRL that regulated PKR degradation by NSs.

### Cullin1 binds to NSs most efficiently and regulates PKR degradation

**Fig 3:**
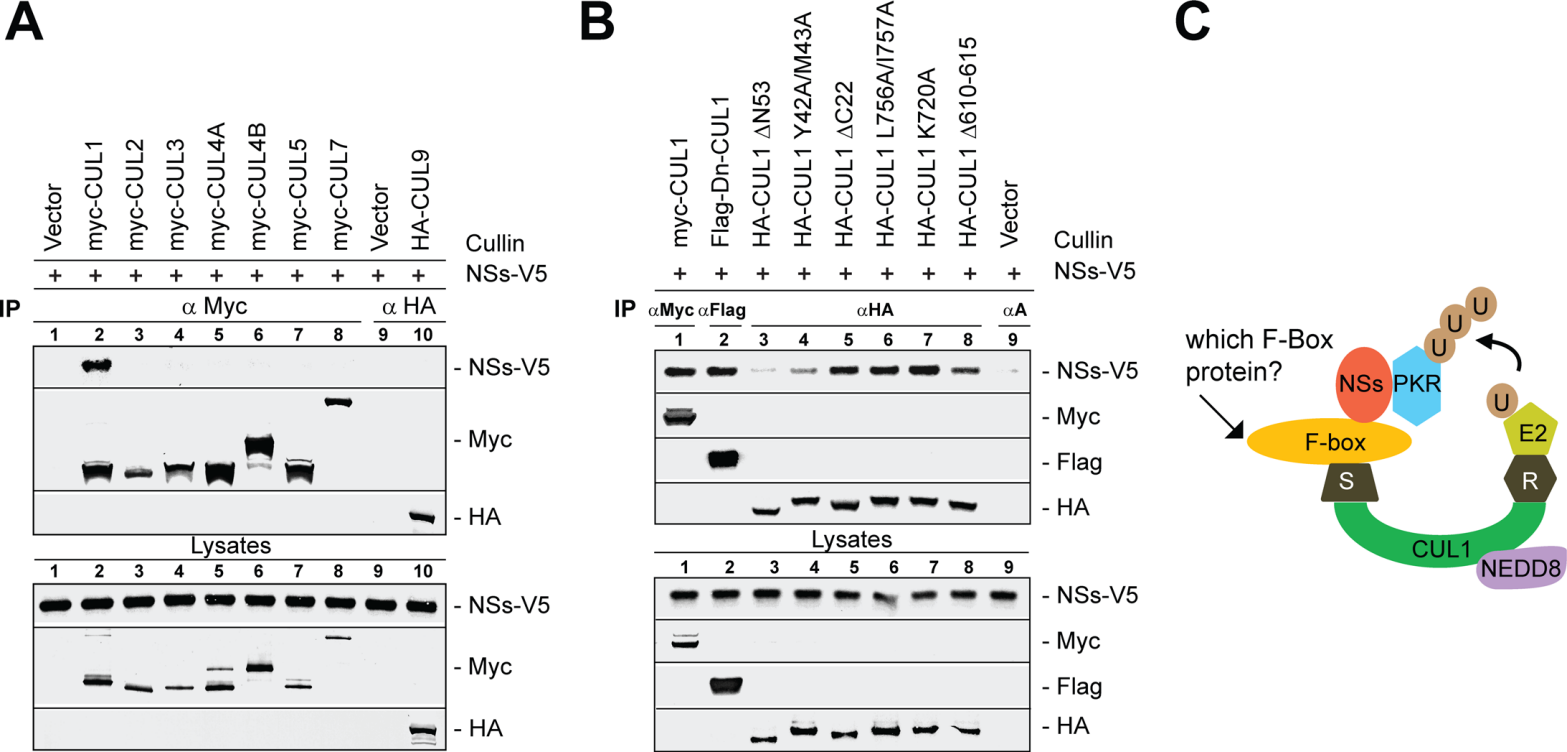
NSs binds to CUL1 most efficiently and regulates PKR degradation. **(A)** Co-immunoprecipitation (Co-IP) of the NSs protein shows that CUL1 bound most efficiently to NSs among the 8 cullin family members, including CUL1 to 3, CUL4A-4B, CUL5, CUL7 or CUL9. Lysates of 293T cells that were transfected to express the vector alone or *myc*-tagged CUL1, -2, -3, -4A, -4B, -5 or -7 or HA tagged CUL9 proteins were combined with rMP-12-NSs-V5 infected cell lysates and immunoprecipitated with the anti-*myc* or anti-HA antibodies. The bound proteins were detected by Western blot analysis as shown in the figure. Lysate controls represent 5% of the lysate used in co-IP. **(B)** NSs protein binding to various CUL1 mutant proteins that were transiently expressed in 293T was determined as described above in A. αA represents IP using all three: HA, *myc* and Flag antibodies in the control **(C)** Tentative model of PKR degradation by SCF complex: CUL1 forms a molecular scaffold to organize the SCF by forming two distinct functional modules. The C terminal domain (CTD) of CUL1 (green) binds to RBX1 (R, grey), that recruits ubiquitin (U, brown) loading E2 enzyme (light green) for catalysis while the N terminus binds to SKP1 (S, grey), which recruits substrate in this case NSs (red)-PKR (blue),) *via* substrate recognizing F-box protein (yellow). PKR is presumably poly-ubiquitinated (UUU, brown) by the E2 enzyme and subjected to proteasome degradation. CUL1 is conjugated with NEDD8 (purple) and is essential for SCF E3 ligase activity.

We hypothesized that NSs targeted PKR degradation by assembling CRL E3 ligase. Thus, we evaluated the binding of NSs to various cullin family members by co-immunoprecipitation (co-IP) assay (Fig 3A). Lysates from 293T cells that were transfected with vector alone or myc-tagged CUL1-3,-4A,-4B, -5 and -7 or HA-tagged CUL9 expression vectors were combined individually with rMP-12-NSs-V5 infected cell lysates and immunoprecipitated with anti-myc or anti-HA antibodies to determine NSs binding to the corresponding CRL. NSs bound to CUL1, but not with the other cullin family members (CUL2,-3,-4A,-4B, -5, -7 or -9). Furthermore, overexpression of dominant negative CUL1, but not other cullin family members, activated PKR in RVFV-infected cells (S2 Fig), thus providing functional significance to the NSs-CUL1 interaction.

CUL1 forms a molecular scaffold to organize CRL1 by forming two distinct modules. The carboxyl (C) terminal domain binds to RBX1, which recruits the ubiquitin-loaded E2 enzymes for catalysis while the amino (N) terminus of CUL1 binds to the adaptor protein SKP1, which recruits the substrate via the substrate recognizing F-box protein (Fig 3C). Using previously characterized CUL1 mutants (see Methods section) that selectively lost their ability to interact with SKP1 or RBX1; we tested for NSs binding by co-IP assay. As shown in Fig 3B, NSs bound to wt-CUL1 (lane 1) and to the CUL1 mutants that have retained their capacity to interact with SKP1 (lanes 2, 5, 6, 7 and 8); in contrast, NSs binding to CUL1 was severely reduced with the mutants CUL1 ΔN53 and CUL1 Y42A/M43A, which cannot bind SKP1 (lanes 3 and 4). Taken together, these data suggest that NSs binds to the CRL1/SCF complex *via* the classical substrate binding motif in the CUL1 subunit, and thus this interaction is most likely facilitated by an F-box (see model in Fig 3C).

### F-box protein FBXW11 binds to NSs and regulates RVFV infection

**Fig 4:**
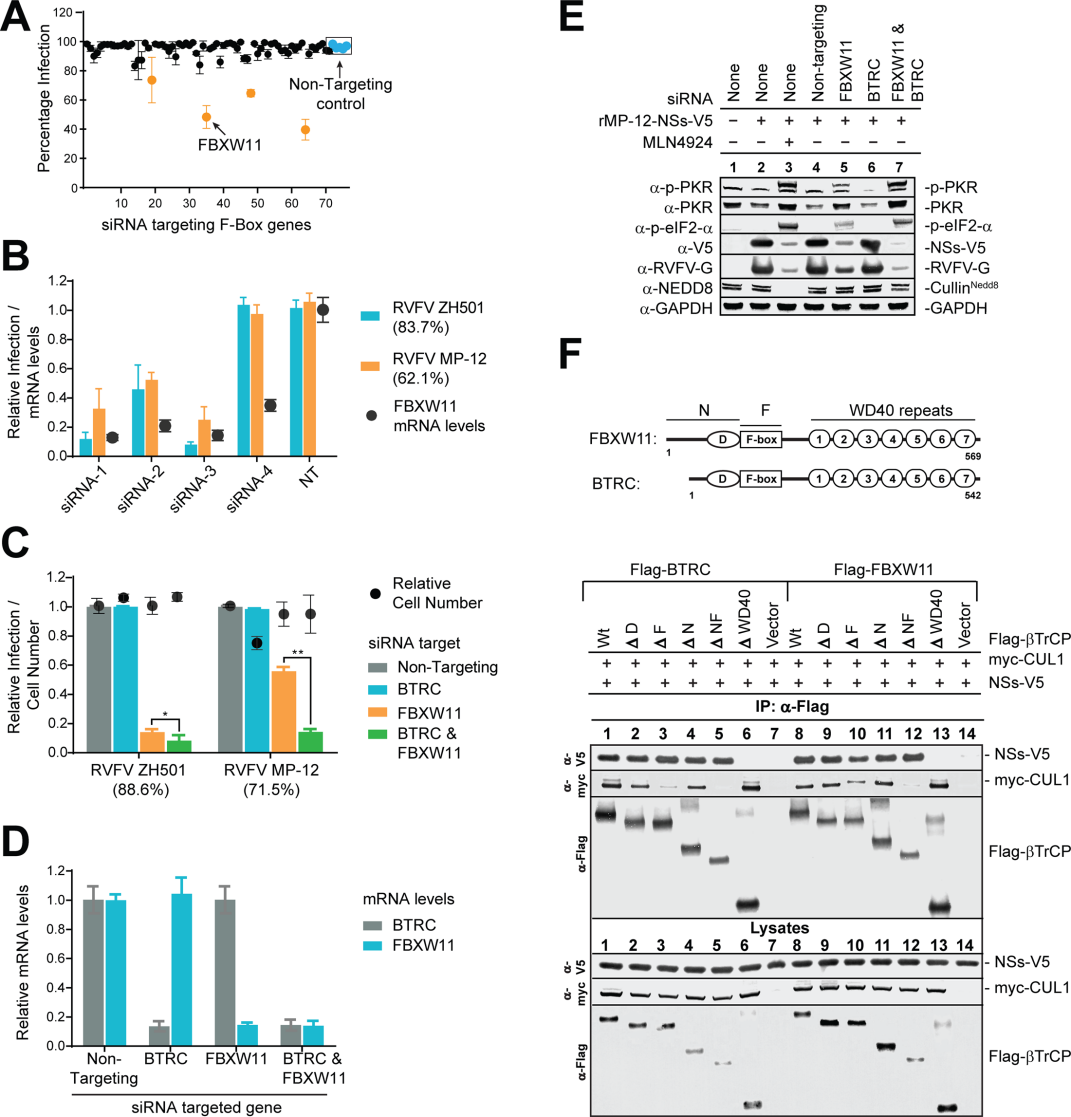
F-box protein FBXW11 binds to NSs and regulates RVFV. **(A)** siRNA screening assay targeting 70 out of 72 human F-box genes to determine RVFV infection inhibitors. HeLa cells were transfected with non-targeting control siRNA or with a pool of four different siRNA targeting each of the 70 F-box genes for 48h followed by incubation with ZH501 (MOI = 1) for 24h. Percentage of infected cells was determined by HCA of N expressing cells. Each data point in panels A to D is an average of three replicates ±SD and is representative of 2 independent experiments. **(B)** Validation of FBXW11 as a true hit from the siRNA screening: Each siRNA from the pool of 4 siRNAs was individually tested for its ability to inhibit RVFV (ZH501 or MP-12) infection and reduce FBXW11 mRNA levels. Infection was quantified by HCA of viral antigen expressing cells as described in A, while mRNA levels were determined by real time PCR. The relative infection or mRNA levels were calculated by normalizing with the values derived from controls cells that were transfected with non-targeting siRNA and infected with the corresponding viruses. The infection rates of control siRNA treated cells are indicated in the brackets next to the virus names. **(C)** FBXW11 is the major homologue of βTrCP gene regulating RVFV infection, but its activity is enhanced by BTRC. Same as in B except siRNA targeting BTRC was used either singly or combined with FBXW11 siRNA. **(D)** Real-Time PCR analysis of BTRC and FBXW11 mRNA levels in HeLa cells that were transfected with the siRNAs are indicated on the X-axis. Relative mRNA levels were derived by normalizing mRNA levels in cells transfected with non-targeting siRNA. **(E)** Western blot analysis shows that βTrCP siRNA knockdown induces PKR activation and inhibition of viral gene expression similar to MLN4924 treated cells. HeLa cells were transfected with different siRNAs as indicated for 48h and then either mock infected or infected with rMP-12-NSs-V5 (MOI = 10, 8h), and were either treated with DMSO or MLN4924 at 2h, PI. Cells lysates were harvested at 8h, PI for Western blot analysis. **(F)** Co-immunoprecipitation (co-IP) assay is consistent with the WD40 domain regulating βTrCP binding to NSs: Top panel, depicts the domain structure of the BTRC and FBXW11 protein which contains D or the Dimerization domain, F or F-box motif, and the seven WD40 repeats. In the bottom panel is data from co-IP assay showing the binding of NSs or CUL1 to the various deletion mutants of BTRC and FBXW11. 293T cell lysates overexpressing myc-CUL1 and the vector alone or flag tagged wildtype βTrCP or deletion mutants of βTrCP were combined with rMP-12-NSs-V5 (MOI = 10, 8h) infected cell lysates and immunoprecipitated with anti-Flag antibody. The bound proteins were detected by Western blot analysis as described in the figure. Lysate controls represent 5% of extract used for co-IP.

We speculated that if NSs-PKR were recruited by an F-box protein, then depletion of the specific F-box protein would prevent PKR degradation, activate PKR, and inhibit RVFV infection. Thus, we used an siRNA screening assay targeting 70 out of the 72 F-box genes characterized in the human genome to determine the specific F-box gene(s) involved in RVFV infection [21,22]. CDRT1 and FBXO49 were not classified as F-box genes during the time this study was initiated and therefore were not included in our siRNA screening assay (refer to gene list in S3 Fig). For this assay, a pool of 4 different siRNAs was used to knockdown each of the 70 F-box genes in triplicate. As shown in Fig 4A, siRNA knockdown of four genes resulted in 30–50% inhibition of ZH501 infection in HeLa cells (marked in orange). Further validation of the hits was achieved by de-convoluting the pool of four siRNAs into individual siRNAs and testing their antiviral activity, cellular cytotoxicity and their ability to decrease target mRNA levels. A hit was considered genuine if two or more siRNAs showed antiviral activity without cytotoxic effects (<15% decrease in cell number). Among the four hits, siRNA’s targeting FBXW11 alone passed these criteria (Fig 4B and 4C). As shown in Fig 4B, 3 out of the 4 individual siRNAs targeting FBXW11 showed greater than 70% knockdown efficiency in mRNA levels and also inhibited viral infection of both MP-12 and ZH501 strains by greater than 50%. The fourth siRNA was able to knockdown FBXW11 mRNA by approximately 70%, however, it did not inhibit viral infection. These data suggest that a higher level (greater than 70%) of FBXW11 mRNA knockdown is required to inhibit RVFV infection. These data were also validated with three additional siRNAs targeting different regions of the mRNA (S4A Fig). Furthermore, antiviral activity due to loss of FBXW11 expression was specific to RVFV infection and was effective in multiple cell lines including 293T and HSAEC (human small airway epithelial cells) primary cells, thus following the same pattern of antiviral activity displayed by MLN4924 treatment (S4B Fig).

*FBXW11* and *BTRC* are two distinct homologues of the *βTrCP* gene and many studies suggest that they are functionally redundant [23]. Thus we determined, whether BTRC partially compensated for loss of FBXW11 expression in RVFV infection. As shown in Fig 4C, combined knockdown of both BTRC and FBXW11 was more potent in suppressing RVFV infection than knockdown of FBXW11 alone. Surprisingly loss of BTRC expression had no effect on RVFV infection and was compensated by FBXW11. The observed antiviral effects were not due to cytotoxicity or non-specific effects of siRNA knockdown (Fig 4D). Thus, FBXW11 plays a dominant role among the two homologues in RVFV infection.

We then tested if knockdown of βTrCP inhibited RVFV infection through the same mechanism as global inactivation of CRL by MLN4924 treatment. This included loss of PKR degradation, PKR activation that resulted in increased expression levels of p-PKR and p-eIF2-α, followed by inhibition of viral gene expression within 8 h of RVFV infection. This pattern of antiviral gene expression was evaluated by Western blot analysis. As shown in Fig 4E, loss of FBXW11 expression alone (lane 5) or FBXW11 and BTRC combined together (lane 7) showed the same gene expression pattern as inactivating CRLs by MLN4924 treatment (lane 3). However the phenotype of FBXW11 knockdown was milder when compared to combined knockdown of BTRC and FBXW11. FBXW11 played a dominant role and loss of FBXW11 expression recapitulated loss of global activity by CRLs during RVFV infection.

Based on these data, we predicted that FBXW11 might be the key factor that recruited NSs-PKR to the SCF complex, and thus examined the NSs-FBXW11 interaction. As shown in Fig 4F (top panel), the βTrCP protein contains the N terminal “D” or protein dimerization domain and the F-box motif that regulates recruitment to the SCF complex *via* binding to SKP1. The seven WD40 repeat motifs in the C terminus together form a beta-propeller like structure and regulate substrate binding. We characterized the NSs-βTrCP interaction by co-IP assay. As shown in Fig 4F (bottom panel), NSs binds to both homologues, BTRC and FBXW11, of βTrCP. Furthermore, WD40 repeats play an essential role in this interaction. As a control, the CUL1 binding to βTrCP mutants was analyzed. As reported previously, F-box mutants (ΔF and ΔNF deletion mutants) reduced the CUL1-βTrCP interaction. Overall, it appears that NSs targets PKR degradation by assembling SCF^FBXW11^.

### SCF^FBXW11^ regulates NSs-mediated PKR degradation

**Fig 5:**
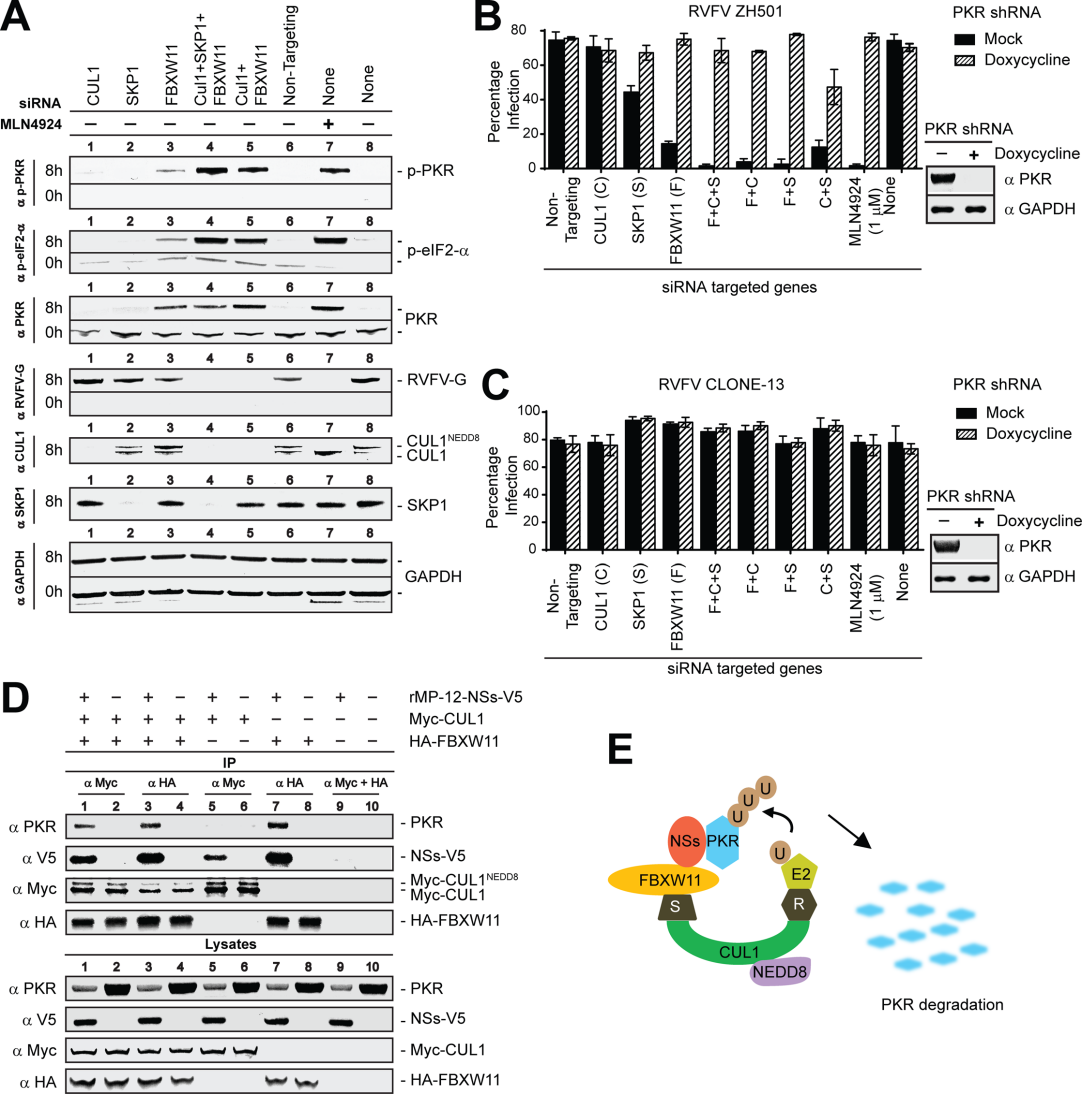
SCF ^FBXW11-NSs^ is the E3 ligase that targets PKR degradation during RVFV infection. **(A)** Western blot analysis to determine the PKR activation and viral gene expression inhibition of rMP-12-NSs-V5 (MOI = 10 for 8h) infected HeLa cells in which the expression of the individual components of the SCF^FBXW11^ E3 ligases were either knocked down singly or in combination with siRNA. **(B)** Evaluating SCF^FBXW11^ regulation of RVFV infection under normal or PKR depleted conditions. The inset on the right is a Western blot showing PKR levels in the HeLa cell line that stably expresses doxycycline inducible PKR shRNA under untreated or doxycyline treated conditions. HeLa cells that were either treated with control DMSO or doxycycline were transfected with siRNA targeting the individual components of SCF^FBXW11^, singly or in combination as indicated in the figure for 48h followed by infection with RVFV ZH501 (MOI = 1 for 24h). The percentage of infected cells was determined by HCA analysis of RVFV N expressing cells. Each data point is an average of 3 replicates ±SD. **(C)** Same as in B except that RVFV Clone 13 (MOI = 3, 24h) was used in infection. **(D)** Co-immunoprecipitation assay showing that PKR is recruited to the SCF^FBXW11^ complex by NSs. 293T cell lysates transfected with empty vector or expression plasmids of *myc*-CUL1 or HA-FBXW11 or *myc*-CUL1 and HA-FBXW11 (as indicated in the figure) were combined with mock infected or rMP-12-NSs-V5 (MOI = 10 for 8h) infected cell lysates and immunoprecipitated with anti-*myc* or anti-HA, or a combination of both. Western blot of lysates represents 5% of the total cellular extracts. **(E)** Model of NSs recruiting PKR to the SCF *via* NSs binding to FBXW11. The symbols R, S, and U represent RBX1, SKP1 and Ubiquitin respectively.

If SCF^FBXW11^ targets PKR degradation, then loss of expression of key components of this complex should inhibit RVFV infection by the same mechanism as global inactivation of CRL by MLN4924 treatment. We therefore examined the status of the PKR activation pathway and viral gene expression by Western blot analysis of mock or rMP-12-NSs-V5 infected HeLa cells in which the individual components of the SCF^FBXW11^ were knocked down by siRNA, either singly or in combination. As shown in Fig 5A, knockdown of FBXW11 in combination with CUL1 and SKP1 (lane 4) or with CUL1 alone (lane 5) resulted in the same gene expression signature as MLN4924-treated cells (lane 7). Knockdown of FBXW11 (lane 3) had a milder effect; whereas, knockdown of CUL1 (lane 1) or SKP1 (lane 2) alone had no effect and was identical to control siRNA treated cells. SiRNA knockdown reduces, but does not completely eliminate, the protein in the cells. Therefore, low residual expression of CUL1 or SKP1, in the presence of endogenous expression levels of FBXW11 was probably sufficient to rapidly degrade PKR. Overall, these data suggest that SCF^FBXW11^ is the primary complex that regulates PKR degradation, and failure to destroy PKR leads to a concomitant increase in p-PKR and p-eIF2-α levels, which suppresses viral protein synthesis.

If this is the case, then depleting PKR in the cell should rescue the viral inhibition resulting from knockdown of the SCF^FBXW11^ complex. We approached this question by using a HeLa cell line that stably expresses a doxycycline-inducible PKR shRNA. We first confirmed by Western blot that doxycycline treatment decreased PKR expression in these cells (Fig 5B, inset). Next we examined the effects of knocking down SKP1, CUL1, FBXW11 alone or in combination, in the presence or absence of PKR, on infection levels of RVFV ZH501 in HeLa cells. As shown in Fig 5B, under normal PKR expression conditions, at saturating infection rates, depletion of CUL1 had no effect on RVFV infection, while SKP1or FBXW11 depletion resulted in greater than 40% or 80% reduction in infection. Moreover, knockdown of FBXW11 in combination with CUL1 and SKP1, individually or in combination, resulted in nearly complete inhibition of RVFV infection, similar to that observed when cells were treated with MLN4924. The infection inhibition by loss of key components of SCF^FBXW11^ was completely reversed by loss of PKR expression as expected. These data support the idea that SCF^FBXW11^ is the primary complex that regulates PKR degradation, and failure to destroy PKR acts to suppress viral protein synthesis and thus viral infection.

To further examine the role of NSs in the degradation of PKR via SCF^FBXW11^ and in the activation of PKR, we repeated the experiment using RVFV Clone13, which encodes a non-functional NSs gene. As shown in Fig 5C, knockdown of any of the components of the SCF^FBXW11^ complex did not inhibit viral infection, which was similar to that observed with MLN4924 treatment. These data strongly suggest NSs regulated PKR activation in conditions when PKR degradation was blocked by inactivation of SCF^FBXW11^.

We speculated that PKR is recruited to the SCF^FBXW11^ complex for degradation by the NSs protein. Thus, we examined the binding between PKR and the SCF^FBXW11^ complex in the presence and absence of NSs protein. As shown in Fig 5D, PKR bound to CUL1 or FBXW11 in the presence of NSs (lanes 1 and 3), but no binding of PKR to CUL1 or FBXW11 was detected in the absence of NSs (lanes 2 and 4). Furthermore, FBXW11 appears to serve as the NSs-PKR adaptor of the SCF complex and a limiting factor for this interaction as well since in the absence of exogenously expressed FBXW11, there was substantially lower amounts of NSs and no PKR detected in the CUL1-bound fraction (lanes 5 and 6). However, similar levels of NSs or PKR were bound to FBXW11, even in the absence of exogenous CUL1 expression (lanes 7 and 8). Taken together, these data provide strong evidence that NSs-FBXW11 serves as the PKR receptor of the SCF^FBXW11^ complex as shown in the model presented in Fig 5E. Furthermore, the protein binding data, combined with siRNA knockdown results, suggests that FBXW11 serves as the limiting factor in the assembly of SCF^FBXW11^ complex.

### Degron sequence of NSs regulates NSs-FBXW11 interaction

**Fig 6:**
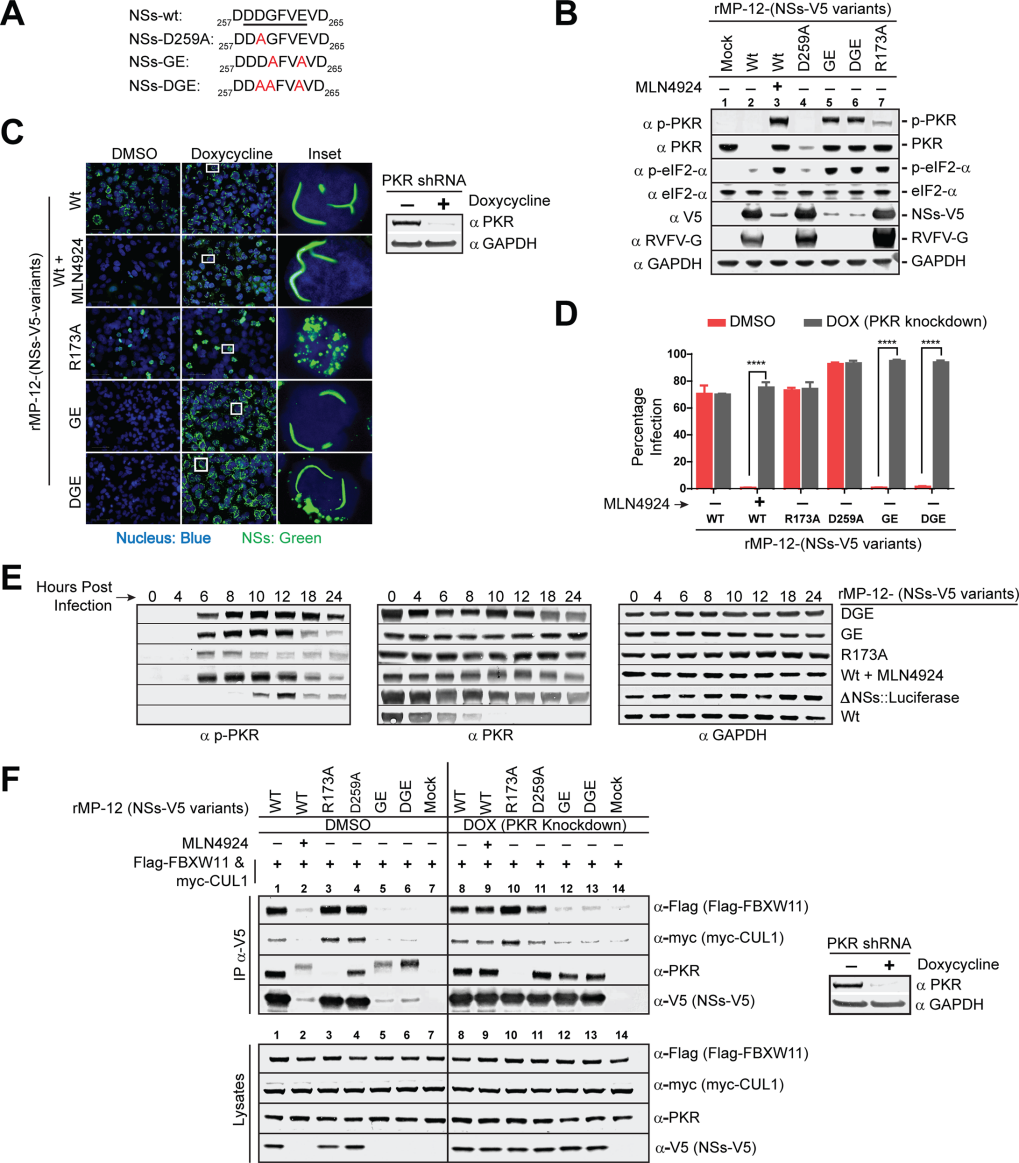
Characterization of the degron/destruction sequence that is essential for NSs-FBXW11 interaction to promote PKR degradation. **(A)** Description of the NSs degron mutants. The six linear amino acid sequence predicted as the degron sequence of the NSs gene is underlined and the alanine substitution mutations are marked in red. The number in the subscript denotes the amino acid residue number of the NSs protein. **(B)** Western blot analysis to determine the PKR activation and viral gene expression status in HeLa cells that were infected with rMP-12 encoding wildtype NSs gene or the NSs mutants as indicated in the figure at MOI = 10 for 8h. **(C)** IFA of NSs (green) filament formation in the nucleus of HeLa cells that were infected with rMP-12 virus expressing wildtype NSs gene or any of the various NSs mutants as indicated in the figure at MOI = 1 for 24h under normal or PKR depleted conditions. The inset shows the magnified image of white box marked in Doxycycline treated cells. Nuclei are stained blue with Hoechst (33342). The panel on the right side is the Western blot showing PKR levels in the HeLa cell line that stable express doxycycline inducible PKR shRNA under untreated or doxycycline treated conditions. **(D)** Same as C, but the percentage of infected cells was determined by HCA analysis of G expressing cells. Each data point is an average of 3 replicates ±SD. **** indicates a P < 0.0001. **(E)** Western blot analysis of p-PKR and PKR expression kinetics in HeLa cells that were infected with rMP-12 virus expressing wildtype NSs gene or any of the NSs mutants as indicated in the figure. GAPDH was used as the loading control. **(F)** Co-immunoprecipitation assay demonstrating binding between NSs mutants and FBXW11 or CUL1. 293T cell lysates transfected with Flag-FBXW11 and myc-CUL1 expression plasmids were combined with mock infected or rMP-12 encoding wildtype or mutant NSs-V5 (MOI = 10 for 8h) under normal or PKR depleted conditions as described in the figure. Lysates were immunoprecipitated with anti-V5 antibodies to test NSs binding to FBXW11, CUL1 and PKR. Western blot of lysates represents 5% of the total cell extracts.

Numerous studies have shown that βTrCP recognizes a 6–9 linear amino acid sequence also known as degron (or destruction) sequence that starts with two acidic amino acid residues followed by a small amino acid (usually glycine) and another acid moiety in the last position. These acidic amino acids and glycine were shown to play an essential role in βTrCP-substrate interaction. We found one degron-like sequence, DDGFVE_263_, in the C-terminal acid-rich region of the NSs protein (Fig 6A). Using a reverse genetics system, we generated several NSs degron mutants and subsequently rescued the mutant viruses. We speculated that rMP-12 encoding NSs mutants that cannot bind to FBXW11 will be non-infectious in HeLa cells due to PKR activity (Fig 4E). We also included in this analysis the rMP-12-NSs-R173A-V5 mutant virus, which was previously described as incapable of binding to PKR [15]. As shown in Fig 6B, NSs-GE and NSs-DGE mutants in lanes 5 and 6, respectively, showed the same gene expression profile as global inactivation of CRLs (lane 3) with potent inhibition of late viral gene expression. On the other hand, the NSs-D259A mutant was similar to wildtype virus. This mutation was probably compensated by the surrounding acidic amino acid residues. Most importantly, NSs-R173A induced PKR activity, as reported previously [19], but was not as pronounced as NSs-GE or DGE mutants and did not inhibit viral gene expression. We next examined if the GE and DGE mutants indeed inhibited infection due to PKR activation by testing virus infectivity in PKR knockdown cells.

A HeLa cell line that stably expresses doxycycline inducible PKR-shRNA was used to evaluate the replication of rMP-12 expressing NSs-GE or DGE mutants under normal or low PKR expression conditions. We first examined NSs gene expression by IFA at 24h PI. As shown in Fig 6C, NSs-GE or DGE mutant viruses showed severe inhibition in NSs gene expression, but in cells treated with doxycycline in which PKR expression is reduced, the NSs expression was restored. The NSs-GE and DGE mutants formed filamentous structures in the nucleus identical to the wildtype NSs (see inset). This pattern of NSs gene expression was also similar to MLN4924 treated and wildtype rMP-12 virus infected cells. In contrast the NSs-R173A mutant, formed mosaic structures in the nucleus, as previously reported [15], and did not show any difference in NSs gene expression under normal or PKR depleted conditions. These data suggested that NSs-GE or DGE mutations, unlike NSs-R173A, did not undergo a conformational change and retained most of the properties of the wildtype NSs in structure and function.

We also examined infection by these mutant viruses by enumerating the percentage of cells that expressed G on the cell surface (a reflection of successful virus egress) using HCA after multiple rounds of infection (Fig 6D). As expected, rMP-12-NSs-GE or DGE expressing mutant viruses showed potent inhibition of infection under normal PKR expression levels, but their infection was completely restored under low PKR expression levels similar to the observations made with MLN4924 treatment of wildtype rMP-12 infected cells. On the other hand, the R173A mutant replicated efficiently irrespective of PKR levels. We next examined the kinetics of PKR activation to understand the NSs mutant’s role in activating PKR.

As shown in Fig 6E, rMP12-NSs-GE or DGE mutants, similar to MLN4924 treated and wildtype virus infected cells, failed to promote PKR degradation and instead caused a steady increase in PKR activity based on p-PKR levels starting from 6 to 18h PI. Although the NSs-R173A mutant blocked PKR degradation, it induced low levels of PKR activation and for a short period of time (6-8h PI). The low p-PKR expression levels were probably not sufficient to severely inhibit NSs-R173A mutant replication. Furthermore, similar to earlier observations (Fig 2C) a functional NSs gene was essential for early PKR activation since induction of p-PKR activity was not observed until the very late stages of infection (10h PI) in the rMP-12ΔNSs::Luci infected cells. Taken together, these data suggest that NSs activates PKR when PKR degradation is blocked. In addition, NSs-R173A is not as effective as the wildtype NSs gene in activating PKR.

We next examined if NSs-GE or NSs-DGE mutant viruses indeed blocked NSs binding to FBXW11. Only under very low conditions of PKR expression did the NSs-GE or NSs-DGE mutants express virus abundantly (Fig 6C). Therefore, HeLa cells stably expressing doxycycline inducible PKR shRNA were used for co-IP assay. Lysates of 293T cells that were transfected with HA-FBXW11 and myc-CUL1 expression plasmids were combined with the lysates of HeLa cells infected with wildtype rMP-12 or rMP-12 mutant viruses expressing NSs -R173A, -D259A, -GE, or -DGE mutants that were either mock treated or treated with doxycycline for 48h prior to start of infection. Cell lysates were immunoprecipated with the V5 antibody to detect NSs binding to FBXW11 and CUL1 (Fig 6F). The NSs mutants, NSs-GE and NSs-DGE expressed abundantly only in HeLa cells that had low PKR expression and did not bind to FBXW11 or CUL1 protein, unlike the wildtype or NSs-D259A or NSs-R173A mutant proteins. Furthermore, NSs-R173A did not interact with PKR similar to previously reported observations [19], while NSs-DGE or NSS-GE mutants bound PKR to the same extent as wildtype NSs. Overall, these data suggest that “DDGFVE” is the degron sequence that regulates the NSs-FBXW11 interaction. NSs-GE and DGE mutants that bind to PKR, but cannot bind to FBXW11, failed to assemble SCF^FBXW11^ to promote PKR degradation. Meanwhile, other unknown NSs activities in the cell (details in Discussion) stimulated PKR activity that induced phosphorylation of eIF2-α, leading to global translational suppression including inhibition of viral protein synthesis.

## Discussion

**Fig 7:**
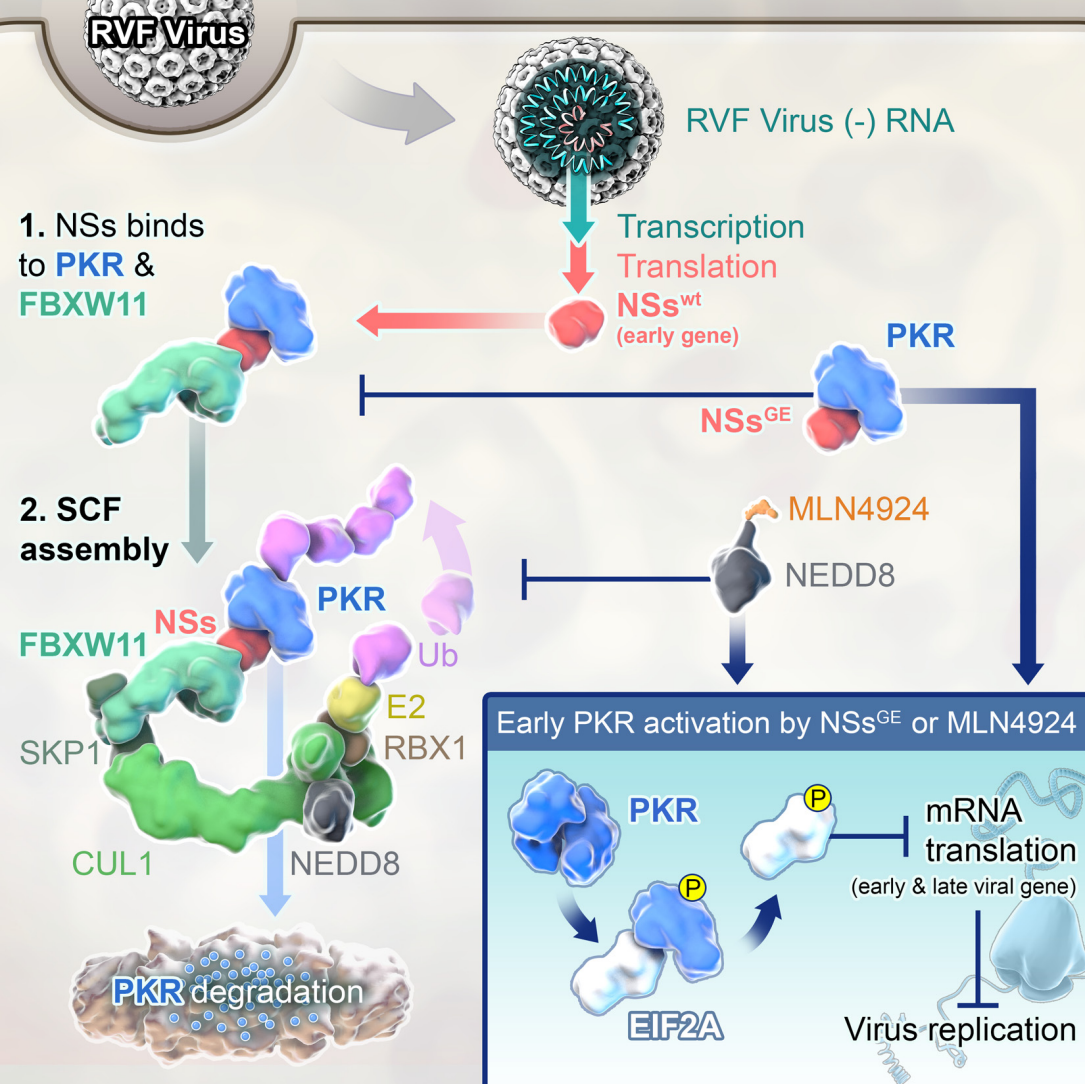
Model illustrating that the inactivation of SCF^FBXW11^ E3 ligase activates PKR and inhibits RVFV infection. NSs, is expressed early during infection and targets PKR proteosomal degradation by assembling SCF (SKP1-CUL1-F-box)^FBXW11^ E3 ligase. The degron sequence in NSs regulates NSs-FBXW11 interaction and is required in the assembly of the SCF^FBXW11-NSs^ complex. NSs also recruits PKR to the SCF^FBXW11-NS^ E3 ligase through an alternate binding site. PKR is mostly ubiquitinated by the E2 ubiquitin conjugating enzyme of the SCF^FBXW11^ E3 ligase and is subsequently targeted for proteosomal degradation. Disassembly or inactivation of SCF^FBXW11-NSs^, which is achieved by the RVFV degron mutant expressing NSs-GE that cannot promote NSs-FBXW11 interaction or by MLN4924 treatment that blocks cullin NEDDylation inhibits PKR degradation. Under these conditions when PKR degradation is blocked, NSs through an as yet not clearly defined mechanism activates PKR. Activated PKR then auto-phosphorylates and subsequently phosphorylates eIF2-α. Accumulation of phosphorylated eIF2-α shuts down ongoing protein synthesis including viral gene translation leading to potent inhibition of RVFV infection.

PKR degradation is one of the numerous strategies that viruses such as RVFV and poliovirus have evolved to counter the PKR antiviral activities, although the specific mechanisms have not been clearly defined previously [13,14,24]. In this study, we show that NSs of RVFV regulates PKR degradation by recruiting PKR to the SCF^FBXW11^ complex (see model in Fig 7). In spite of our best efforts, we could not demonstrate ubiquitinated PKR. This could be due to a combination of low endogenous expression levels of PKR and high proteasome activity.

Early degradation of PKR by NSs was absolutely essential during RVFV infection. Failure to do so resulted in PKR activation and impaired late viral protein production. MLN4924 that blocked PKR degradation by inactivating SCF/CRL activity or rMP-12 NSs-GE and NSs-DGE mutants that cannot assemble SCF^FBXW11-NSs^ E3 ligase, induced robust PKR activation and potent inhibition of late viral protein synthesis (model in Fig 7). These inhibitory effects of PKR could be complemented by loss of PKR expression. This is similar to the antagonism of PKR activity by the E3L protein of poxviruses [25]. Under normal infection conditions, E3L blocks PKR access to double stranded (ds)-RNA, thereby preventing PKR activation. A E3L deletion mutant vaccinia virus (ΔE3L) shows potent activation of PKR activity and inhibition of late viral protein production. Loss of PKR expression rescued infection by an E3L deficient virus. However, unlike poxviruses, NSs and not dsRNA, is the trigger for early PKR activation during RVFV infection.

We show that NSs has dual antagonistic functions. NSs activates PKR but only under conditions when NSs mediated PKR degradation is blocked. An NSs deficient virus (rMP-12 ΔNSs::Luci) that cannot degrade PKR did not induce early PKR activation (Figs 2B, 2C and 6E). This lack of early PKR activation was not due to a delay in virus replication kinetics, since no differences in the viral gene expression kinetics were observed with the NSs deficient virus when compared to a wildtype virus during the first cycle of replication (Fig 2C). This also excludes dsRNA as a possible trigger since viral replication kinetics during the first cycle of infection was not different between NSs deficient and wildtype viruses. Furthermore, unlike positive-stranded RNA or DNA viruses, dsRNA accumulation was not detected in negative-stranded RNA virus infected cells [26]. However during late stages of infection, an NSs deficient virus activated PKR, mostly due to induction of the interferon pathway, whose effects are felt during the second cycle of replication. PKR activation during late stages of infection (10-12h PI) had little impact on the first cycle of infection because by then a majority of the viral protein is made (Fig 2C). Lastly, transient transfection of NSs expression was sufficient to activate PKR upon MLN4924 treatment. Collectively, these data suggest that NSs is both essential and sufficient to activate PKR when NSs mediated PKR degradation is selectively blocked.

But how does NSs activate PKR? It is possible that the cellular stress responses triggered by NSs could activate PKR. Many different types of cellular stress, including oxidative stress and DNA damage response induced by NSs could potentially activate PKR [27,28]. Furthermore, NSs suppression of host transcription by RNA pol I and II could activate PKR. Indeed actinomycin D (small molecule inhibitor of transcription) treatment of NSs-deleted RVFV mutant infected cells can activate PKR; although, the exact mechanism has not been defined [13]. Furthermore, direct binding of NSs to PKR may activate PKR; although, the fact that an NSs-R173A mutant that did not bind to PKR still activated PKR, albeit less efficiently, does not favor this hypothesis (Fig 6). Ongoing studies are focused on addressing the mechanism of NSs-mediated PKR activation.

Another interesting and important observation made in this study is the dominant role of FBXW11, over its homologous counterpart BTRC, in the regulation of PKR degradation. *BTRC* and *FBXW11* are two homologues of the *βTrCP* gene that are biochemically indistinguishable and play a redundant role in substrate degradation [29]. At least 35 substrates of βTrCP, with well-described roles in controlling cell cycle and signal transduction pathway, have been described, including CDC25, which is a positive regulator of the cell cycle, β-catenin, and IκB (an inhibitor of NFκB) [23,30]. Despite their important roles in regulating a wide range of cellular functions, the BTRC gene knockout mice (BTRC^-/-^) did not affect viability or show any gross tissue abnormalities other than impaired spermatogenesis in male mice [31,32]. BTRC^-/-^ MEFs did not accumulate any of the well-known cellular targets of βTrCP. Additionally, siRNA knockdown of FBXW11 by greater than 77% in BTRC^-/-^ MEFs was required to accumulate βTrCP substrates. Thus these data suggest that BTRC and FBXW11have a redundant role *in vivo*. This property makes FBXW11 an attractive therapeutic target, since knockout of FBXW11 homologue alone may prevent RVFV infection without negatively affecting other immune responses regulated by βTrCP in mice. Recently, the development of floxed FBXW11^flox/flox^ has been reported and future studies with these mice will help to answer these questions [33].

Why is FBXW11 more effective than BTRC in NSs mediated PKR degradation? The dominant role played by FBXW11 in the RVFV infection model is not due to differences in the binding properties between NSs and βTrCP homologues, since both proteins (BTRC and FBXW11) bind with similar affinities to NSs *via* their WD40 domain (Fig 4F). The lack of redundancy could be explained by differences in the cellular localization of the two homologues and the substrate being targeted. A recent study showed that the more abundant splice variants of BTRC and FBXW11 were expressed in nuclear and cytoplasmic compartments, respectively [34]. PKR is predominantly expressed in the cytoplasm; whereas, NSs expression is observed both in the cytoplasm and nucleus during early stages of infection. NSs in the cellular compartment may recruit FBXW11 targeting PKR proteasomal degradation. NSs in the nuclear compartment may not have access to either PKR or FBXW11. A recent study shows that NSs binds to the p62 subunit of the TFIIH complex *via* the ΩXaV motif (where Ω = Trp or Phe; X = any amino acid; a = Asp or Glu; and V = Val) present in NSs [35]. The ΩXaV motif (FVEV_264_) partially overlaps with the degron sequence (DDGFVE_263_) of NSs. As a result, p62, which is a nuclear protein, could compete with βTrCP to bind to the same site of NSs in the nucleus making it inaccessible to βTrCP. Furthermore, NSs was shown to promote p62 degradation during early stages of infection by assembling the SCF^FBXO3^ through NSs-FBXO3 interaction [36]. Therefore it is possible that the cytoplasmic fraction of NSs assembled SCF^FBXW11^; whereas, the nuclear fractions of NSs assembled SCF^FBXO3^ to target PKR and p62 degradation, respectively. Future work will test these speculations.

In summary, we have shown that NSs assembles CRL to target degradation of host factors whose loss of expression is critical to overcome innate immune responses during RVFV infection. Future studies using an animal model of RVF will be required to address the *in vivo* relevance of these observations.

## Materials and Methods

### Cells and viruses

HeLa, 293T, HepG2, mouse embryonic fibroblasts (MEFs) and Vero E6 cells were obtained from the American Type Culture Collection (ATCC) and were maintained under humidified conditions at 37°C, 5% CO_2_ in Dulbecco's Modified Eagle Medium supplemented with 10% fetal bovine serum (FBS, Life Technologies). HSAECs (Lonza, USA) were maintained in Ham’s F12 medium supplemented with nonessential amino acids, pyruvate, β-mercaptoethanol and 10% fetal calf serum (FCS). RVFV strains ZH548/MP-12 (MP-12) and ZH501 were obtained from The Salk Institute’s Government Services Division and from Dr. Michael Turell (USAMRIID), respectively. MP-12 was derived from a virulent strain of RVFV (ZH548) and is attenuated in its virulence due to several nucleotide mutations in its genome, but encodes a functional NSs gene [13,14] and can be handled safely under biosafety level (BSL)-2 laboratory conditions. ZH501 is the wild-type strain of RVFV and is fully virulent, requiring use under BSL-3 laboratory conditions. Clone 13 virus was a gift from Dr. Friedemann Weber (Philipps-University Marburg, Germany). Clone 13 was isolated from the 74HB49 strain of RVFV from a human case and encodes a non-functional NSs gene due to a large internal frame deletion that removed 69% of the ORF. VEEV (1CSH3), LASV (Josiah) and MARV (Ci67) were obtained from the USAMRIID collection. All viruses were propagated in Vero E6 cells. Virus-containing supernatants were clarified by centrifugation at 12,000 x *g* for 30 min prior to storage at -80°C. All virus stock titers were determined by plaque assay on Vero E6 cells as previously described [37].

### Virus infections

RVFV ZH501 and VEEV infections were performed in a BSL-3 lab, and MP-12 infections were performed in BSL-2 lab. Work with infectious filoviruses or arenaviruses were performed in BSL-4 labs. All infection assays that required enumeration of infected cells based on immunofluorescence assay (IFA) of viral antigen expressing cells were optimized as described previously [16,17]. The infection conditions were optimized such that at a minimal MOI, greater than 50% of cells expressed viral antigen after multiple rounds of infection. This is to ensure that irrespective of the stage of the viral life cycle targeted by the small molecule or siRNA, the infection rates after multiple rounds of infections are decreased. For all the assays described here, HeLa cells were permissive to infection and were seeded at 20,000 cells per well a day prior to infection in a 96-well plate (black well, clear and flat bottom from Greiner) and infected with virus at the required MOIs in a total volume of 100 μl. Inocula were removed 1h post virus incubation, unless stated otherwise, washed one time with 1x PBS, and replaced with an equal amount of fresh medium. Infection was allowed to proceed for the specified duration of time as indicated in the figure legends of each experiment, followed by fixation in 10% neutral buffered Formalin (Sigma) for one day for BSL-3/4 viruses or 15 min for BSL-2 viruses. Cells were then subjected to immunofluorescence assay (IFA) as described below.

### Immunofluorescence assay

IFA of viral antigen expression was carried out as described previously [17]. If permeabilization was required then Formalin-fixed cells were treated with 0.1% (v/v) Triton-X 100 for 15 min at room temperature prior to blocking. Otherwise Formalin fixed cells were directly blocked and incubated with primary antibody (at 1:1000 dilution) prepared in blocking buffer containing 3% BSA/PBS for 1h at room temperature (RT). Then cells were washed three times in PBS followed by incubation with the corresponding fluorescent secondary antibody for 1h at RT. Cells were washed in PBS to remove excess antibody. During the last wash, cell nuclei and cytoplasm labelling reagents, which include Hoechst 33342 (Life Technologies) and HCS Deep Red (Life Technologies), respectively, were added to the wash solution at a 1:10,000 dilution. The following antibodies, 4D4, R3-1D8-1-1a, 1A4A-1, 6D8-1, MBG II 9G4-1, L52-161-6 (from USAMRIID hybridoma bank) were used to detect RVFV G, RVFV N, VEE envelope 2 (E2) protein, MARV GP and Lassa virus GP, respectively.

For measuring RVFV infections (MOI = 1, 24 h) that involved multiple rounds of infection, 4D4 antibody that detects RVFV Gn was used with MP-12 virus. Since the 4D4 antibody does not detect Gn expression of ZH501, N expression was used for measuring infection by ZH501 and clone 13 specifically for most of the study except for Figs 1B and S1A where Gc antibody (5G3) was used. Since the Gc antibody was only available in limited amounts, it was used only for these figures to show that under the conditions of infections used N or G expression did not significantly affect the interpretation of the data. G was used to represent Gn expression throughout the manuscript except in Figs 1B and S1A where Gc was used. As described in the introduction, Gn and Gc form heteromers and are produced by proteolytic processing of the precursor GPC. Therefore Gn or Gc detection for these two figures did not change the interpretation of the data. For NSs-V5 tagged protein and Flag-FBXW11, anti-V5 and anti-Flag (Sigma) antibodies were used. The following secondary antibodies were used: Alexa 488-conjugated goat anti-mouse secondary antibody, Alexa 568-conjugated goat anti-rabbit antibody or Alexa 647-conjugated goat anti-rabbit (Life Technologies) at 1:1000 dilutions.

### High content image analysis

Confocal images were collected using a Leica TCS-SP5 confocal/multiphoton microscope with a 40x oil objective. High-content quantitative imaging data were acquired and analyzed on an Opera confocal reader (model 3842 [Quadruple Excitation High Sensitivity] or model 5025; PerkinElmer) at two exposures using a 10x air objective. High-content image (HCI)-based analysis (HCA) was accomplished within the Opera or Columbus environment using standard Acapella scripts as described previously [17,38]. Briefly, images of Hoechst stained nuclei were used to draw nuclear boundaries and to count cells, while images of Cell Mask Deep Red stained cells were used for drawing cell boundaries. Images of viral antigen expression were used for enumerating percentage of infected cells.

### Concentration response curve analysis

A 10-point dose-response curve (1/3 fold serial dilution from 30 μM) assay was used for dose response curve analyses. Each concentration of the hit compound was tested in triplicate in a 96-well plate format. Cells were treated with the indicated concentrations of MLN4924 for 1h prior to incubating cells with MP-12 or ZH501 at MOI = 1. One hour post virus incubation, cells were washed and replaced with compound containing media. Twenty-four hours post-infection, cells were fixed and subjected to IFA followed by HCA to evaluate the percentage of RVFV infection by enumerating the viral antigen expressing cells. Relative infection inhibition was determined by the ratio of the percentage of infected cells in compound treated cells with mock treated (0.5% DMSO) and RVFV infected cells. Data were analyzed using the non-linear regression formula: log (inhibitor) vs. response–variable slope (4 parameters) in GraphPad Prism 6. The IC_50_, defined as the effective concentration resulting in a 50% inhibition of infection, was used to evaluate compound activity. The relative cell number was determined by normalizing the cell number of compound treated + virus-infected cells with mock treated (0.5% DMSO) + virus-infected cells.

### Co-immunoprecipitiation (co-IP) assay and antibodies

Cells infected with rMP-12 (MOI = 10 for 8 h) encoding wildtype NSs-V5 or its mutants served as sources of NSs protein. These lysates were combined with cell lysates expressing the protein of interest. Transient overexpression by plasmid DNA transfection was carried out using Lipofectamine 2000 (Life Technologies). For co-IP assay, cells in a 12 well dish were lysed in IP lysis buffer (Life Technologies, cat # 87787) supplemented with complete protease inhibitor cocktail (Roche) at 4°C for 20 min and clarified at 14,000 x *g* for 15 min at 4°C. Ten percent of the cleared lysate was kept aside as a lysate control, while the remaining lysate was incubated with 2 μg of the indicated antibody and 25 μl of washed protein A/G plus agarose beads and allowed to rotate mildly for 3h at 4°C. The beads were then washed three times in lysis buffer and one last wash with high salt PBS containing 300 mM NaCl. The bound proteins were eluted by boiling in Laemmli sample buffer, and separated by SDS-PAGE, followed by transfer to a polyvinylidene (PVDF) membrane. The blots were incubated for 1h at RT or overnight at 4°C with the indicated primary antibodies. After three washes, the blots were incubated with the appropriate alkaline phosphatase (AP)-conjugated secondary antibodies (GE Healthcare) according to the manufacturer's recommendations. The blots were washed and developed by a Western blotting detection system (GE Healthcare). For direct protein analyses, cells in 12-well dishes were lysed in 100 μl of 1X Laemmli sample loading buffer and boiled for 5 min at 95°C. Equal amounts of samples were used for Western blot analysis as described above. The following antibodies were used for Western blot analysis or immunoprecipitation (when required): T446 phosphorylated PKR (Abcam), PKR (BD Transduction), S52 phosphorylated EIF2α (Cell Signaling), EIF2α (Cell Signaling), TFIIH-p62 subunit (Santa Cruz), NEDD8 (Cell Signaling), CUL1 (Santa Cruz), SKP1 (Cell Signaling) and alkaline phosphatase (AP) conjugated V5 antibody (Life Technologies). The antibodies to HA, V5, Flag and Myc were from Sigma.

### Generation of doxycycline inducible PKR shRNA expressing stable cell line

HeLa cells were transduced with lentivirus expressing doxycycline (Sigma) inducible human PKR shRNA (cat # V2THS_170555, GE Healthcare) encoding the antisense sequence 5’-TTTATCTCTGATGTATCTG-3’ and selected in Puromycin (1 μg/ml, from Sigma) containing media. The HeLa stable cell line can be induced with doxycyline (1 μg/ml) to express PKR shRNA.

### Mutations and virus rescue

The plasmid DNA for recombinant MP-12 (rMP-12) virus rescue including the S segment encoding plasmid DNA in which the NSs ORF was replaced by a luciferase gene was a kind gift from Dr. Shinji Makino (Univ. of Texas Medical Branch, US). Mutations in the NSs gene, including the addition of a V5 tag to the gene were introduced using Q5 Site-Directed Mutagenesis Kit (New England Biolabs) by following the manufacturer’s recommendations. The virus was rescued as described previously [20]. Briefly, subconfluent BSR-T7/5 cells were co-transfected with an S-genome RNA expression plasmid, such as pProT7-S(+) or its variants encoding NSs mutations and a mixture of pPro-T7-M(+), pPro-T7-L(+), pT7-IRES-vN, pCAGGS-vG, and pT7-IRES-vL using TransIT-LT1 (Mirus Bio Corporation). The culture medium was replaced with fresh medium 24h later. At 5 days post-transfection, the culture supernatants were collected, clarified and inoculated onto VeroE6 cells. The supernatant of infected VeroE6 cells at 2–3 days PI were aliquoted and stored at -80°C.

### siRNA screening targeting human F-box genes

The 70 siRNAs targeting human F-box genes were cherry picked from the Dharmacon ON-TARGETplus SMARTpool siRNA Library—Human Ubiquitin Conjugation Subset 2 or 3 (list in the supplementary data S4 Fig). The smart pool contained 4 different siRNAs targeting individual gene and were screened against RVFV ZH501 in triplicate at a concentration of 15 nM in a 96-well plate format using optimized assay conditions as described previously [9]. Briefly, HeLa cells (12,500 cells per well) were reverse transfected using HiPerfect (Qiagen) transfection reagent (0.6 μl per well) in a total volume of 100 μl per well in 96-well plates following the manufacturer’s instructions. As a positive control, 3 wells of each plate were transfected with non-targeting siRNAs. Mock infected cells were treated as negative controls. On the following day cells were washed and replaced with fresh media. Forty-eight hours post transfection, cells were subjected to RVFV infection (MOI = 1, 24h). The percentage of infected cells was determined using HCA analysis of N expressing cells. Data were represented as the average ±SD.

### Plasmid constructs and siRNAs

pcDNA3-FLAG-UBC12 (C111S) was a gift from Dr. Tetsu Kamitani [18]. The cullin expression plasmids, pcDNA3-myc3-CUL1,-2,-3, -4A,- 4B, -5, -7 or HA2-PARC/CUL9 were gifts from Dr. Yue Xiong [39–41] (Addgene # 19896, 19892, 19893, 19951, 19922, 20695, 19895 and 20937 respectively). The cullin mutant expression plasmids, pcDNA3-HA-CUL1 ΔC22, L756A/I757A, K720A, Y42A/M43A, Δ610–615 or ΔN53 (19942, 19938, 19939, 19940, 19941 and 19950 respectively) were gifts from Dr. Yue Xiong [42,43]. The dominant negative (Dn) cullin expression plasmids pcDNA3-CUL1-3, 4A, 4B and CUL5 were a gift from Dr. Wade Harper [21] (Addgene plasmid # 15818, 15819, 15820, 15821 and 15822 respectively). pcDNA3-HA-FBXW11 was a gift from Dr. Yi Sun [44]. pcDNA3- Flag-BTRC/FBXW11-Wt, ΔN, ΔF, ΔD, ΔNF and ΔWD40 constructs were a gift from Dr. Tomoki Chiba [45].

The binding properties of the CUL1 mutant proteins with SKP1 or RBX1 were as follows: the wildtype CUL1 contains 776 amino acid (aa) residues of which the N terminal 249 aa regulate its binding to SKP1, while the remaining 527 aa recruit RBX1 [21,42,43].The dominant negative Dn-CUL1 (truncated protein expressing the first 452 aa) or CUL1Δ610–615 cannot bind to RBX1, but retains interaction with SKP1. CUL1-ΔN53 or Y42A/M43A mutants, cannot bind to SKP1, but retain their binding to bind to RBX1. Lastly, the CUL1-ΔC22, L756A/I757A, or the NEDDylation defective mutant K720A can bind to both SKP1 and RBX1.

NSs-V5 gene expression plasmid was generated by sub-cloning the PCR amplified product from the pProT7-S plasmid expressing NSs-V5 gene into pcDNA3.1 vector. The siRNAs targeting BTRC (cat# S17109) was from Ambion while FBXW11 (cat # J-003490-07) CUL1 (cat # J-004086-08) and SKP1 (cat # J-003323-15-0002) were from GE Healthcare.

pcDNA3-FLAG-UBC12 (C111S) was a gift from Dr. Tetsu Kamitani (Wada, H., Yeh, E.T. and Kamitani, T., 2008, J Biol Chem, 275, 17008–15). Dominant negative (Dn) Cullin expression plasmids pcDNA3-CUL1-3, 4A, 4B and CUL5 were a gift from Dr. Wade Harper (Jin, J., Ang, X.L., Shirogane, T. and Wade Harper, J. 2005. Methods Enzymol, 399, 287–309.) (Addgene plasmid # 15818, 15819, 15820, 15821 and 15822 respectively). pcDNA3-HA-FBXW11 was a gift from Dr. Yi Sun (Zhao, Y., Xiong, X. and Sun, Y. 2011. Mol Cell, 44, 304–16).

The siRNA’s-1, -2 and -3 targeting FBXW11 were from Ambion with cat# S23485, S23486 and S23487 respectively.

### Quantitative real-time PCR

Relative changes in RVFV RNA levels or mRNA levels of host genes were determined by qRT-PCR on an ABI Prism 7900HT sequence detection system using RNA UltraSense one-step kit and TaqMan probes (Life Technologies). Total RNA was extracted using the RNeasy Plus minikit (Qiagen). Fifty nanograms of RNA was used per qRT-PCR reaction. The target gene mRNA or viral RNA expression levels were normalized to the PPIB housekeeping gene. Relative expression levels were determined using the comparative threshold cycle (*C*_*T*_) method [46]. The taqman probe sequences used for RVFV RNA have been described previously [47].

### Statistical methods

The student Paired t test was used to determine P values. **** indicated P<0.0001.

## Supporting Information

S1 Fig MLN4924 is a potent inhibitor of RVFV Infection. **(A-B)** HCA performance when compared to plaque assay. HeLa cells seeded in 96 well plate were infected with mock or increasing MOI of the virulent RVFV ZH501 (A) strain or the vaccine strain RVFV MP-12 (B) for 24h. Cells were then subjected to IFA to detect RVFV-G to measure virus infection by HCA of G expressing cells, while the cell supernatants were collected for plaque assay to determine the plaque forming units. **(C)** Overexpression of a dominant negative UBC12 mutant (that blocks NEDDylation), in HeLa cells inhibited RVFV infection specifically: HeLa cells transiently expressing vector or Flag-UBC12(C111S) mutant for 24h were infected with ZH501(MOI = 1, 24h) or VEEV(MOI = 0.1, 24h). The percentage of infected cells was determined by HCA viral antigen expressing cells. **(D)** Time of MLN4924 addition assay shows that compound addition could be postponed as much as 5h PI without effecting its antiviral activity: HeLa cells were incubated with MP-12 virus (at time = 0h) and MLN4924 was added at 1 μM concentration at the indicated time points below X-axis. Infection was stopped at 13h PI and subjected to IFA to determine the percentage of cells expressing G. The average infection rate of mock treated and MP-12 infected cells was 72.3%. **(E)** MLN4924 inhibits viral protein expression from 6-8h PI: kinetics of percentage of RVFV- N, G (determined by IFA of permeabilized cells) or S-G (G expression on cell surface as determined by IFA of non-permeabilized cells) expressing cells at different time points as indicated on the X-axis, post MP-12 (MOI = 10) incubation with HeLa cells. Cells were either treated with control (DMSO) or MLN4924 (1 μM) at 2h PI. **(F)** MLN4924 inhibited viral RNA levels of MP-12 virus from 5-7h PI: HeLa cells were either treated with control (DMSO) or MLN4924 (1 μM) and either mock infected (time = 0h) or infected with MP-12 virus for the time points indicated in the figure. RNA was quantified by real time -PCR. Relative fold change in RNA copy number was calculated from RNA levels at 0h of infection, which was considered as 1. **(G)** MLN4924 treatment of RVFV infected cells could be postponed to 5h PI to observe the decrease in viral RNA synthesis at 7h PI: HeLa cells mock infected or incubated with MP-12 virus (time = 0h) at 10 MOI were either treated with control DMSO or MLN4924 at 1, 3 or 5h PI. Cells were harvested at the indicated time points on the X-axis to quantify viral RNA levels. Relative RNA levels were calculated as in G. All data values described in this figure except in A, is an average of 3 replicates ±SD and is representative of 2 or more experimental repeats.
(TIF)

S2 Fig NSs Binding to CUL1 Regulates PKR Degradation. Western blot analysis demonstrating the induction in p-PKR expression levels in RVFV infected cells expressing dominant negative (dn) CUL1: HeLa cells that were transiently transfected to overexpress control vector or wildtype CUL1 or dominant negative CUL1, -2, -3, -4A, -4B or 5A were either mock infected or infected with MP-12 for 8h. GAPDH expression served as loading control.
(TIF)

S3 Fig List of the F-box genes that were used in siRNA screening assay. List of F-box genes that were used in siRNA screening for antiviral activity against RVFV infection.
(TIF)

S4 Fig FBXW11 regulation of RVFV infection. **(A)** Additional confirmation with siRNAs from a different vendor (Ambion) that shows that RVFV infection is inhibited when >70% of FBXW11 mRNA knockdown is achieved by siRNA. Infection was quantified by HCA of viral antigen expressing cells, while mRNA levels were determined by real time PCR. Relative infection or mRNA levels were calculated by normalizing with the values derived from controls cells that were transfected with non-targeting siRNA and infected with the corresponding viruses. The infection rates of control siRNA treated cells are indicated in the brackets next to the virus names. **(B)** siRNA mediated FBXW11 knockdown inhibited RVFV ZH501 infection in 293T and HSAEC cells.
(TIF)

S1 Text Plasmid constructs and siRNAs used in the Supporting Information. (PDF)
