# Supplementary figures and images for: Protein Kinase R Degradation Is Essential for Rift Valley Fever Virus Infection and Is Regulated by SKP1-CUL1-F-box (SCF)FBXW11-NSs E3 Ligase
Source: PLoS Pathog. 2016 Feb 2;12(2):e1005437. doi: 10.1371/journal.ppat.1005437 (PMC4737497; doi:10.1371/journal.ppat.1005437)

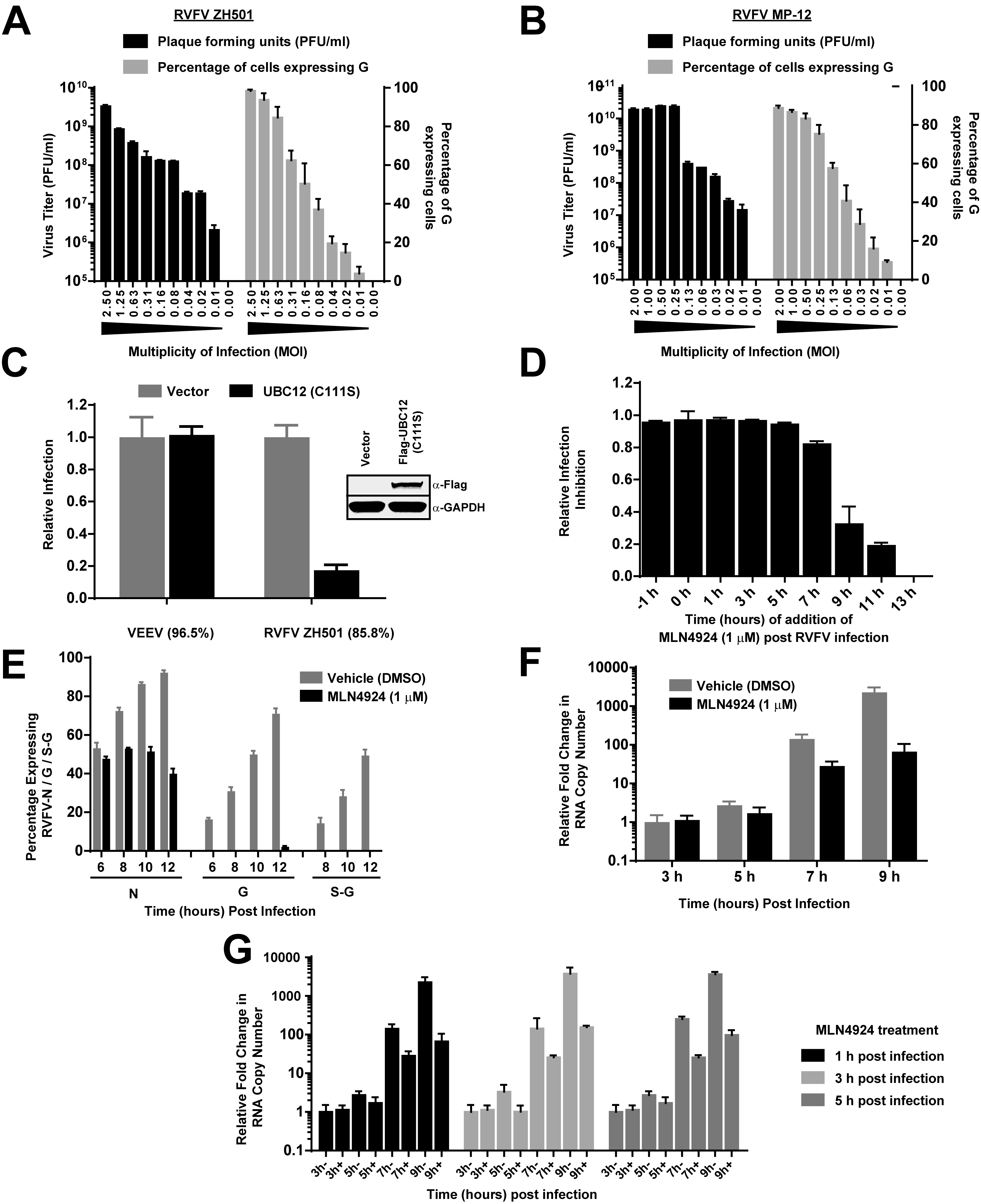

Supplement: S1 Fig — (A-B) HCA performance when compared to plaque assay. HeLa cells seeded in 96 well plate were infected with mock or increasing MOI of the virulent RVFV ZH501 (A) strain or the vaccine strain RVFV MP-12 (B) for 24h. Cells were then subjected to IFA to detect RVFV-G to measure virus infection by HCA of G expressing cells, while the cell supernatants were collected for plaque assay to determine the plaque forming units. (C) Overexpression of a dominant negative UBC12 mutant (that blocks NEDDylation), in HeLa cells inhibited RVFV infection specifically: HeLa cells transiently expressing vector or Flag-UBC12(C111S) mutant for 24h were infected with ZH501(MOI = 1, 24h) or VEEV(MOI = 0.1, 24h). The percentage of infected cells was determined by HCA viral antigen expressing cells. (D) Time of MLN4924 addition assay shows that compound addition could be postponed as much as 5h PI without effecting its antiviral activity: HeLa cells were incubated with MP-12 virus (at time = 0h) and MLN4924 was added at 1 μM concentration at the indicated time points below X-axis. Infection was stopped at 13h PI and subjected to IFA to determine the percentage of cells expressing G. The average infection rate of mock treated and MP-12 infected cells was 72.3%. (E) MLN4924 inhibits viral protein expression from 6-8h PI: kinetics of percentage of RVFV- N, G (determined by IFA of permeabilized cells) or S-G (G expression on cell surface as determined by IFA of non-permeabilized cells) expressing cells at different time points as indicated on the X-axis, post MP-12 (MOI = 10) incubation with HeLa cells. Cells were either treated with control (DMSO) or MLN4924 (1 μM) at 2h PI. (F) MLN4924 inhibited viral RNA levels of MP-12 virus from 5-7h PI: HeLa cells were either treated with control (DMSO) or MLN4924 (1 μM) and either mock infected (time = 0h) or infected with MP-12 virus for the time points indicated in the figure. RNA was quantified by real time -PCR. Relative fold change in RNA copy number w [file ppat.1005437.s001.tif]

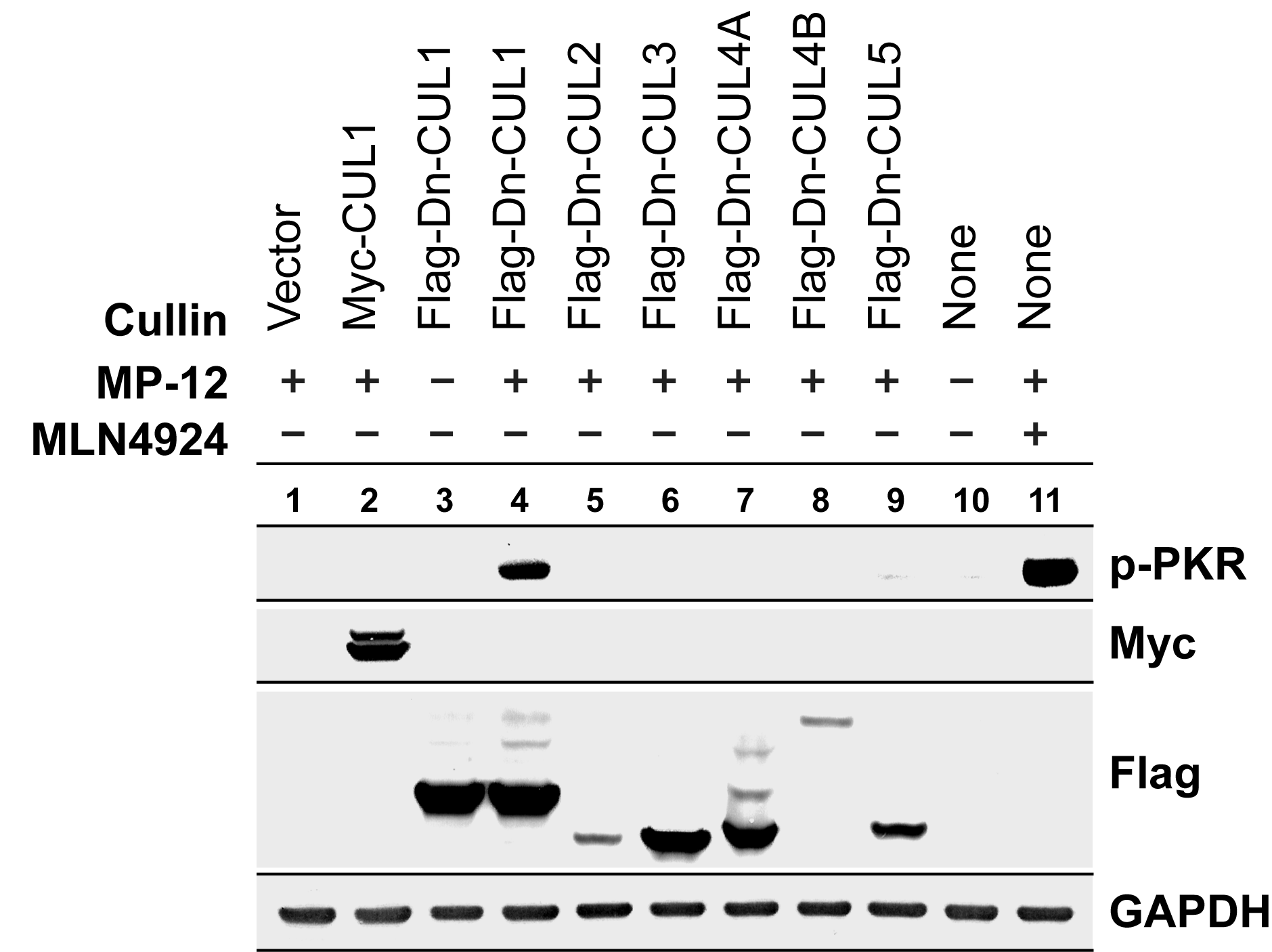

Supplement: S2 Fig — Western blot analysis demonstrating the induction in p-PKR expression levels in RVFV infected cells expressing dominant negative (dn) CUL1: HeLa cells that were transiently transfected to overexpress control vector or wildtype CUL1 or dominant negative CUL1, -2, -3, -4A, -4B or 5A were either mock infected or infected with MP-12 for 8h. GAPDH expression served as loading control. (TIF) [file ppat.1005437.s002.tif]

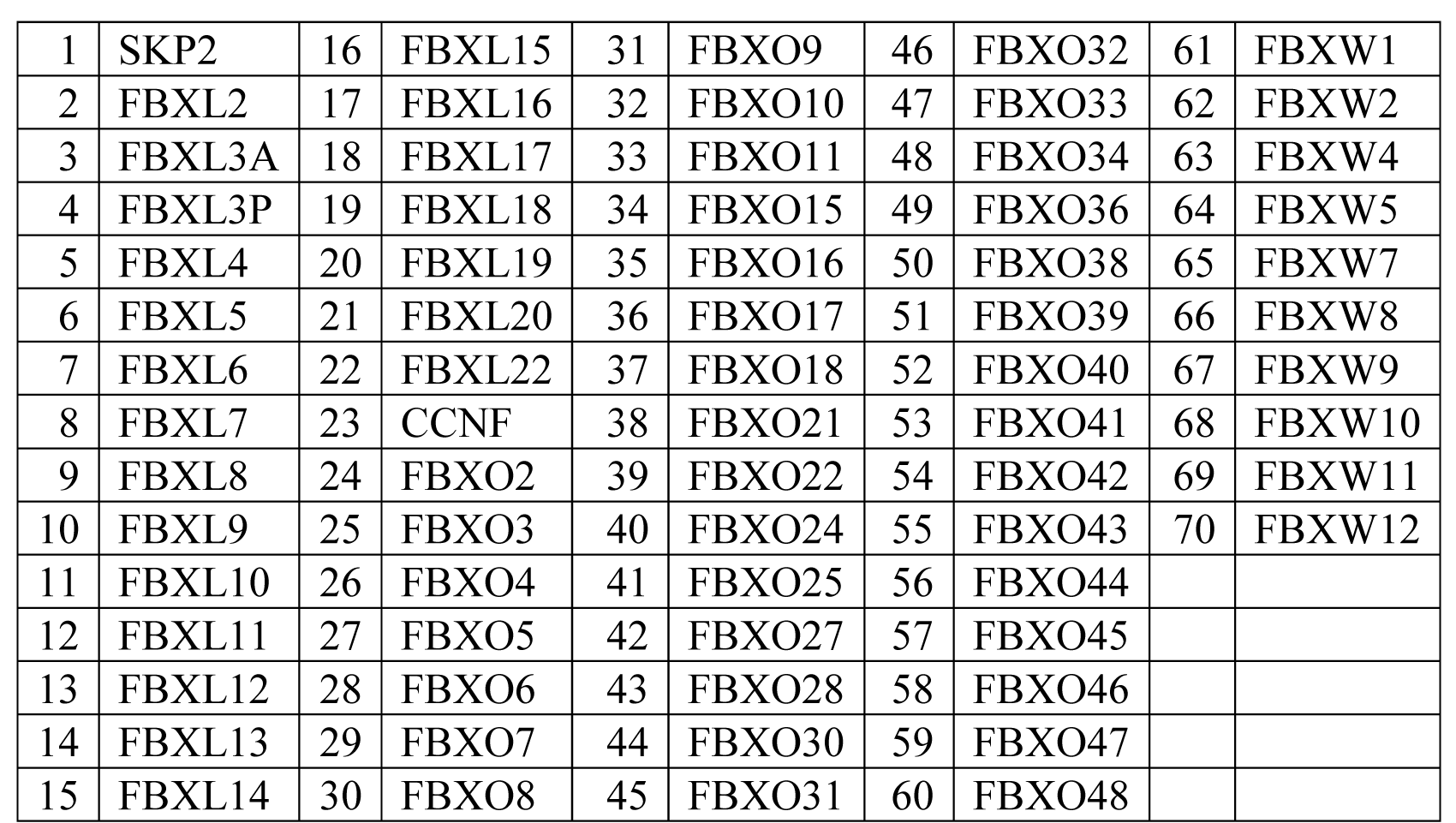

Supplement: S3 Fig — List of F-box genes that were used in siRNA screening for antiviral activity against RVFV infection. (TIF) [file ppat.1005437.s003.tif]

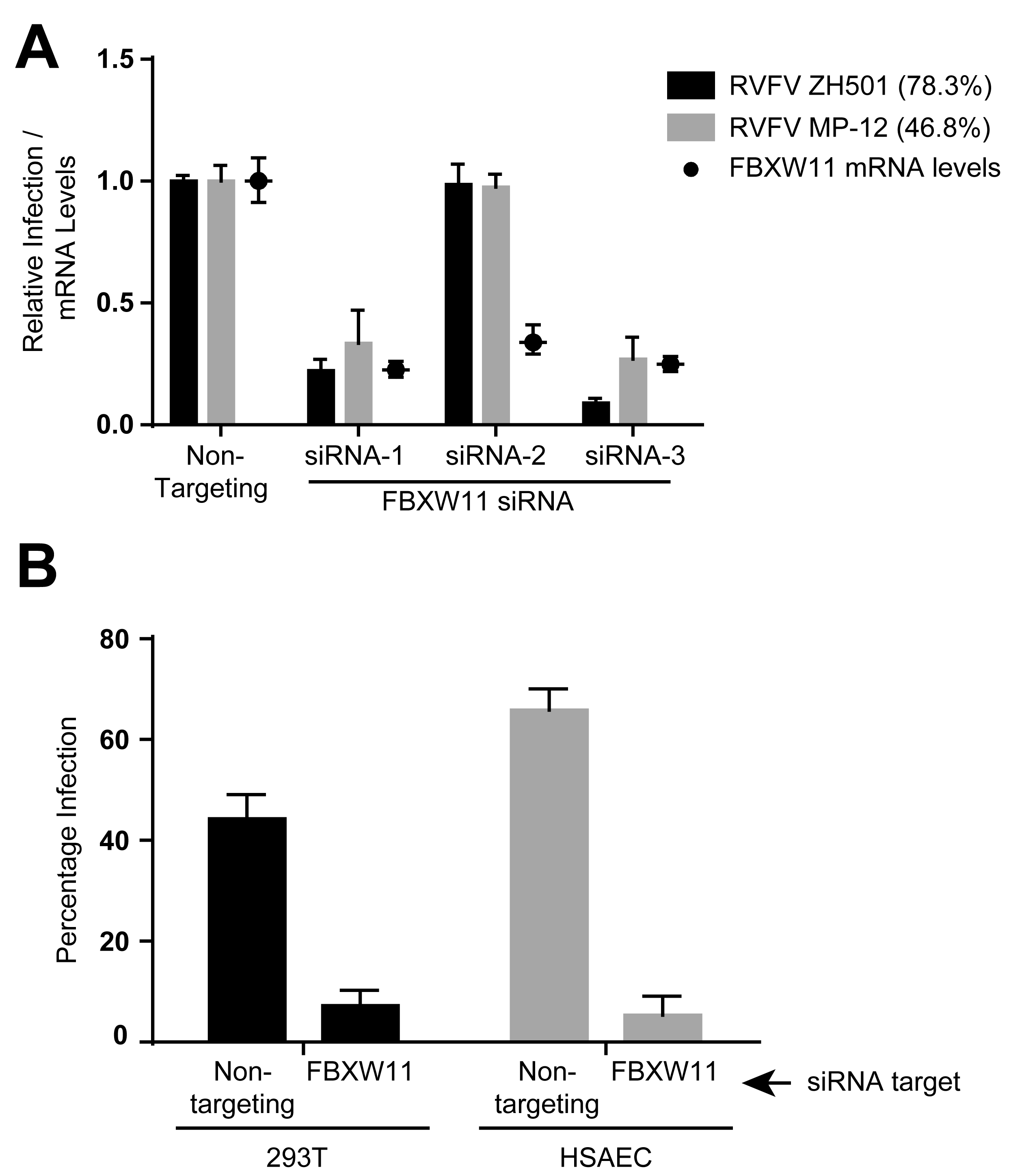

Supplement: S4 Fig — (A) Additional confirmation with siRNAs from a different vendor (Ambion) that shows that RVFV infection is inhibited when >70% of FBXW11 mRNA knockdown is achieved by siRNA. Infection was quantified by HCA of viral antigen expressing cells, while mRNA levels were determined by real time PCR. Relative infection or mRNA levels were calculated by normalizing with the values derived from controls cells that were transfected with non-targeting siRNA and infected with the corresponding viruses. The infection rates of control siRNA treated cells are indicated in the brackets next to the virus names. (B) siRNA mediated FBXW11 knockdown inhibited RVFV ZH501 infection in 293T and HSAEC cells. (TIF) [file ppat.1005437.s004.tif]
