# Supplementary material for: Protein Kinase R Degradation Is Essential for Rift Valley Fever Virus Infection and Is Regulated by SKP1-CUL1-F-box (SCF)FBXW11-NSs E3 Ligase
Source: PLoS Pathog. 2016 Feb 2;12(2):e1005437. doi: 10.1371/journal.ppat.1005437 (PMC4737497; doi:10.1371/journal.ppat.1005437)
Supplement: S1 Text — (PDF) [file ppat.1005437.s005.pdf]

The plasmid pcDNA3-FLAG-UBC12 (C111S) was a gift from Dr. Tetsu Kamitani (Wada, H., Yeh, E.T. and Kamitani, T., 2008, J Biol Chem, 275, 17008-15). Dominant negative (Dn) Cullin expression plasmids pcDNA3-CUL1-3, 4A, 4B and CUL5 were a gift from Dr. Wade Harper (Jin, J., Ang, X.L., Shirogane, T. and Wade Harper, J. 2005. Methods Enzymol, 399, 287-309.) (Addgene plasmid # 15818, 15819, 15820, 15821 and 15822 respectively). pcDNA3-HA-FBXW11 was a gift from Dr. Yi Sun (Zhao, Y., Xiong, X. and Sun, Y. 2011. Mol Cell, 44, 304-16). The siRNA's-1, -2 and -3 targeting FBXW11 were from Ambion with cat# S23485, S23486 and S23487 respectively.
